# Sieving carbons promise practical anodes with extensible low-potential plateaus for sodium batteries

**DOI:** 10.1093/nsr/nwac084

**Published:** 2022-05-05

**Authors:** Qi Li, Xiangsi Liu, Ying Tao, Jianxing Huang, Jun Zhang, Chunpeng Yang, Yibo Zhang, Siwei Zhang, Yiran Jia, Qiaowei Lin, Yuxuan Xiang, Jun Cheng, Wei Lv, Feiyu Kang, Yong Yang, Quan-Hong Yang

**Affiliations:** Nanoyang Group, State Key Laboratory of Chemical Engineering, School of Chemical Engineering and Technology, Tianjin University, Tianjin 300072, China; Nanoyang Group, Joint School of National University of Singapore and Tianjin University, International Campus of Tianjin University, Fuzhou 350207, China; Haihe Laboratory of Sustainable Chemical Transformations, Tianjin 300192, China; State Key Laboratory of Physical Chemistry of Solid Surfaces, Collaborative Innovation Center of Chemistry for Energy Materials and Department of Chemistry, College of Chemistry and Chemical Engineering, Xiamen University, Xiamen 361005, China; Nanoyang Group, State Key Laboratory of Chemical Engineering, School of Chemical Engineering and Technology, Tianjin University, Tianjin 300072, China; Haihe Laboratory of Sustainable Chemical Transformations, Tianjin 300192, China; State Key Laboratory of Physical Chemistry of Solid Surfaces, Collaborative Innovation Center of Chemistry for Energy Materials and Department of Chemistry, College of Chemistry and Chemical Engineering, Xiamen University, Xiamen 361005, China; Nanoyang Group, State Key Laboratory of Chemical Engineering, School of Chemical Engineering and Technology, Tianjin University, Tianjin 300072, China; Nanoyang Group, Joint School of National University of Singapore and Tianjin University, International Campus of Tianjin University, Fuzhou 350207, China; Haihe Laboratory of Sustainable Chemical Transformations, Tianjin 300192, China; Nanoyang Group, State Key Laboratory of Chemical Engineering, School of Chemical Engineering and Technology, Tianjin University, Tianjin 300072, China; Haihe Laboratory of Sustainable Chemical Transformations, Tianjin 300192, China; Nanoyang Group, State Key Laboratory of Chemical Engineering, School of Chemical Engineering and Technology, Tianjin University, Tianjin 300072, China; Haihe Laboratory of Sustainable Chemical Transformations, Tianjin 300192, China; Shenzhen Key Laboratory for Graphene-Based Materials, Engineering Laboratory for Functionalized Carbon Materials, Tsinghua Shenzhen International Graduate School, Tsinghua University, Shenzhen 518055, China; Nanoyang Group, State Key Laboratory of Chemical Engineering, School of Chemical Engineering and Technology, Tianjin University, Tianjin 300072, China; Haihe Laboratory of Sustainable Chemical Transformations, Tianjin 300192, China; Shenzhen Key Laboratory for Graphene-Based Materials, Engineering Laboratory for Functionalized Carbon Materials, Tsinghua Shenzhen International Graduate School, Tsinghua University, Shenzhen 518055, China; State Key Laboratory of Physical Chemistry of Solid Surfaces, Collaborative Innovation Center of Chemistry for Energy Materials and Department of Chemistry, College of Chemistry and Chemical Engineering, Xiamen University, Xiamen 361005, China; State Key Laboratory of Physical Chemistry of Solid Surfaces, Collaborative Innovation Center of Chemistry for Energy Materials and Department of Chemistry, College of Chemistry and Chemical Engineering, Xiamen University, Xiamen 361005, China; Shenzhen Key Laboratory for Graphene-Based Materials, Engineering Laboratory for Functionalized Carbon Materials, Tsinghua Shenzhen International Graduate School, Tsinghua University, Shenzhen 518055, China; Shenzhen Key Laboratory for Graphene-Based Materials, Engineering Laboratory for Functionalized Carbon Materials, Tsinghua Shenzhen International Graduate School, Tsinghua University, Shenzhen 518055, China; State Key Laboratory of Physical Chemistry of Solid Surfaces, Collaborative Innovation Center of Chemistry for Energy Materials and Department of Chemistry, College of Chemistry and Chemical Engineering, Xiamen University, Xiamen 361005, China; Nanoyang Group, State Key Laboratory of Chemical Engineering, School of Chemical Engineering and Technology, Tianjin University, Tianjin 300072, China; Nanoyang Group, Joint School of National University of Singapore and Tianjin University, International Campus of Tianjin University, Fuzhou 350207, China; Haihe Laboratory of Sustainable Chemical Transformations, Tianjin 300192, China

**Keywords:** sodium-ion batteries, sieving carbons, low-potential plateau, pore entrance diameter, pore surface area

## Abstract

Non-graphitic carbons are promising anode candidates for sodium-ion batteries, while their variable and complicated microstructure severely limits the rational design of high-energy carbon anodes that could accelerate the commercialization of sodium-ion batteries, as is the case for graphite in lithium-ion batteries. Here, we propose sieving carbons, featuring highly tunable nanopores with tightened pore entrances, as high-energy anodes with extensible and reversible low-potential plateaus (<0.1 V). It is shown that the tightened pore entrance blocks the formation of the solid electrolyte interphase inside the nanopores and enables sodium clustering to produce the plateau. Theoretical and spectroscopic studies also show that creating a larger area of sodiophilic pore surface leads to an almost linearly increased number of sodium clusters, and controlling the pore body diameter guarantees the reversibility of sodium cluster formation, producing a sieving carbon anode with a record-high plateau capacity of 400 mAh g^–1^. More excitingly, this approach to preparing sieving carbons has the potential to be scalable for modifying different commercial porous carbons.

## Teaser

This work vividly images an ideal configuration of practical carbon anodes for high-energy sodium-ion batteries, which greatly challenge lithium-ion batteries in the use of large-scale energy storage.

## INTRODUCTION

Carbon materials are probably the most promising anode candidates for commercial alkali metal-ion batteries because of their unique potential to deliver reversible low-potential charge/discharge plateaus (LPPs) for high energy density [1–4]. However, the design and development of carbon anodes has long experienced a tortuous path, due to the complicated interfacial electrochemistry and electrode chemistry [5], which is seen in the history of lithium-ion batteries (LIBs) [6]. The graphite anode offers a successful paradigm in LIBs. The ultrasmall interlayer spacing of graphite serves as a sub-nanoscopic sieve and only allows bare lithium ions to enter, thereafter enabling the formation of low-stage graphite intercalation compounds and LPPs (<0.1 V vs. Li^+^/Li) [7,8]. Unfortunately, graphite has an inferior electrochemical performance in sodium-ion batteries (SIBs), which have recently captured widespread attention as a sustainable supplement to LIBs for large-scale stationary energy storage [9–11].

Indeed, the slow commercialization of SIBs is largely due to the lack of practical high-energy carbon anodes that can play a similar role to the one played by graphite in LIBs [12,13]. Most recently, it has been proposed that the LPP-related sodium storage process can be attributed to the formation of sodium clusters inside ultrasmall nanopores of non-graphitic carbons, especially the anthracite-derived soft carbons and carbohydrate-derived hard carbons [9,14–16], which suggests a way for the design of non-graphitic carbon anodes to produce and further extend the LPP [17–19]. Porous carbons (PCs) with open entrances accessible to gas adsorbates are totally free of any LPP and only deliver sloping charge/discharge curves [20–22]. Their large surface area is also accessible to electrolytes and induces severe decomposition to form a thick solid electrolyte interphase (SEI) [22,23]. Solvated sodium ions are preferentially adsorbed on the negatively charged surface of the carbon anodes to form an interfacial electric double layer (IEDL), and it is this IEDL that largely determines the interfacial electrochemistry of carbon anodes [24]. Therefore, before designing carbon anodes for superior sodium storage, it is necessary to understand the basic science on how to limit the formation of an undesired IEDL inside nanopores and to initiate sodium clustering inside nanopores to produce an LPP, and how to reversibly cluster more sodium ions to extend the LPP.

Herein, we propose sieving carbons (SCs) as high-energy anodes for practical SIBs with extensible and reversible LPPs (<0.1 V vs. Na^+^/Na), featuring highly tunable nanopores with a tightened pore entrance. In this regard, PCs are used as precursors since they possess sufficient nanopores to potentially accommodate sodium clusters, although they originally do not initiate the sodium clustering. We show that carefully controlling the pore entrance diameter (PED) of PCs (<0.4 nm) helps screen out solvated sodium ions and enables the formation of sodium clusters and the emergence of LPPs. Using spectroscopic and theoretical studies, an approximately linear correlation between the specific surface area (SSA) of pore bodies in SCs and the plateau capacity is revealed, leading to a record-high plateau capacity of 400 mAh g^–1^. We also show that a pore body diameter (PBD) with an upper limit (∼2.0 nm) guarantees the reversibility of the plateau. This way of preparing SCs has the potential to be scalable for modifying commercial PCs into practical anodes, paving the way for the rapid commercialization of SIBs.

## RESULTS AND DISCUSSION

### Sieving solvated sodium ions by tightening the pore entrance

**Figure 1.**
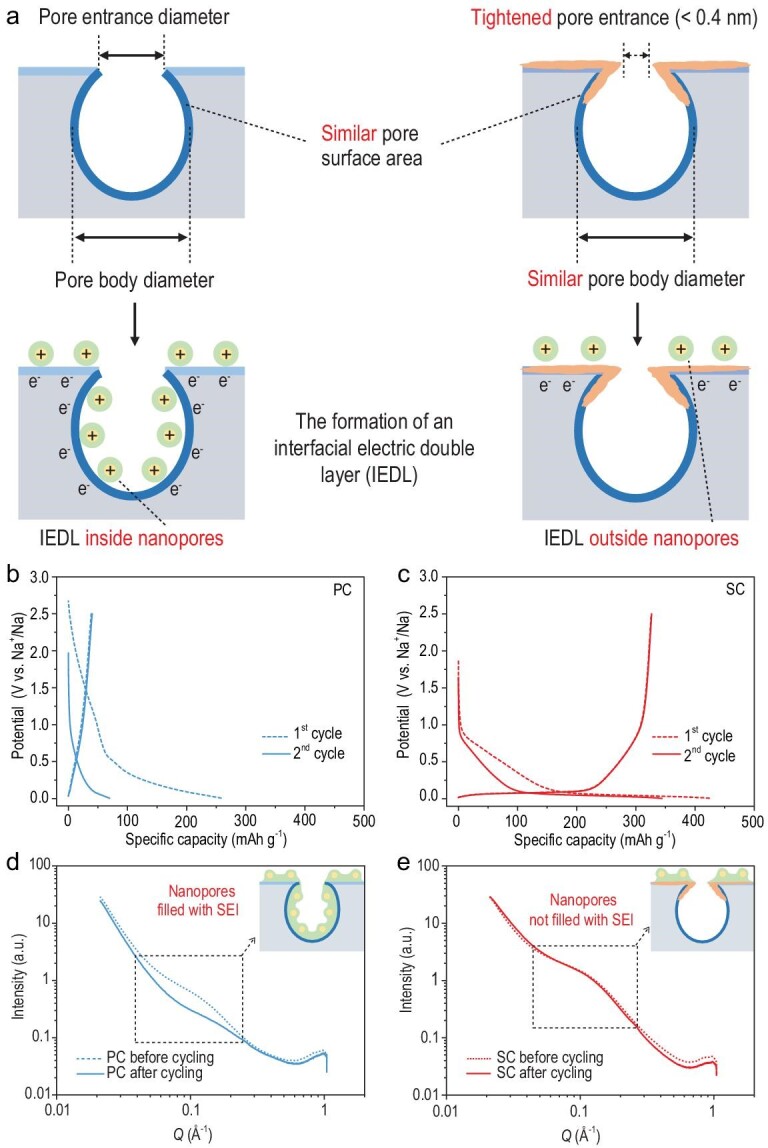
Tightening the pore entrance and regulating the interfacial electrochemistry. (a) Schematic showing the control of the nanopores of a typical porous carbon (left) to produce the target sieving carbon (right), and the comparison between their different IEDLs. PyC, the solvated shell, and Na^+^ are drawn as brown irregular strips, green circles and yellow solid circles with a positive sign, respectively. (b and c) Charge/discharge curves for the first two cycles, at a current density of 50 mA g^–1^, of (b) PC and (c) SC anodes. (d and e) SAXS patterns of (d) PC and (e) SC anodes before and after (dashed line) five full cycles at a current density of 50 mA g^–1^. Inset: the relative location of the SEI to the nanopores. The SEI is a green irregular shape with yellow solid circles (sodium ions) inside.

We used a controlled chemical vapor deposition (CVD) of methane on a range of commercial PCs (with open porosity) to tighten their pore entrances, while maintaining similar PBDs and pore surface areas, in order to produce SCs with smaller PEDs (Figs 1a and S1). In order to precisely control the PED, experimental conditions were carefully studied to avoid excess deposition outside the pores or aimless plugging of the nanopores [25–27]. As a result, a pyrolysis temperature (900°C) and methane concentration (10 mL min^–1^) were used, and only the deposition time was changed. In these conditions, the methane is able to diffuse into accessible pores whose PEDs are larger than a methane molecule. Then methane is adsorbed on the pore walls around the pore entrance, and experiences sequential pyrolysis and polymerization, finally depositing on the pore walls to gradually decrease the PED (Fig. S1). We deduce that the tightened pore entrance restrains the formation of an undesired SEI inside the nanopores, and the electrochemically active pore surface will be protected and available for the formation of sodium clusters.

An activated carbon fiber with large N_2_ adsorption (SSA: ∼1358 m^2^ g^–1^) has abundant nanopores, while after modification of the pore entrance so that it is classified as an SC, has little adsorption (SSA: ∼0 m^2^ g^–1^), indicating its PED is smaller than 0.4 nm (Fig. S2, Table S1) [28–31]. Small angle X-ray scattering (SAXS) was used to investigate the nanopores that cannot be detected by N_2_ adsorption due to geometrical constraints (Fig. S3, Table S2). Signals in the intermediate *Q* range represent carbon porosity [32], and there is only a slight difference between the SC and PC. The calculated SSAs and average PBDs of SC (1298 m^2^ g^–1^ and 2.0 nm respectively) based on SAXS are very close to those of PC (1313 m^2^ g^–1^ and 2.0 nm respectively) (Note S1). It is worth mentioning that the pyrolytic carbon (PyC) from methane is mainly deposited around the pore entrance to reduce its size rather than directly coating the surface of PC [25], as proven by the Raman spectra, with two excitation wavelengths of 532 nm and 325 nm (Figs S4 and S5), and the high-resolution transmission electron microscopy (HRTEM) images (Fig. S6). Moreover, the graphite interlayer spacing and surface chemistry are almost identical for PC and SC (Figs S7–S9, Table S1).

SC has a remarkably better electrochemical performance compared to PC (Fig. 1b and c; Figs S10 and S11). PC shows only a sloping charge/discharge curve with an extremely low initial Columbic efficiency (ICE) of 15% and reversible specific capacity of 39 mAh g^–1^. In sharp contrast, SC has a high ICE of 77% and a reversible capacity of 328 mAh g^–1^, most of which comes from the long LPP. Since the deposited PyC gives only a very low specific capacity without a plateau, we conclude that controlling the PED is the major reason for the appearance of the plateau.

We then investigated the difference in the spatial distributions and compositions of the SEI in cycled PC and SC anodes to show the exact impact of the PED on the interfacial electrochemistry. *Ex-situ* SAXS patterns of the PC anode before and after cycling show the disappearance of the broad peak in the intermediate *Q* range, implying the filling of the nanopores with SEI. In contrast, the nanopore signal of the SC anode remains almost unchanged after the formation of the SEI, which is similar to the conclusion obtained in the sucrose-derived hard carbon anode [33], which shows that the SEI is mainly formed outside the nanopores (Fig. 1d and e). In addition, X-ray photoelectron spectroscopy (XPS) results (Figs S12 and S13) show that the oxygen content of the SC anode (23 at%) is much lower than that of the PC anode (34 at%), suggesting a thinner and organic-depleted SEI. After argon ion etching for 20 mins, the oxygen content of the SC anode decreased to 3 at% while the carbon content increased to 80 at%, consistent with the SAXS results. All these results confirm that controlling the PED regulates the interfacial electrochemistry, which agrees with the significantly lower resistance of Na^+^ diffusion across the SEI (*R_SEI_*) of the SC anode shown by the electrochemical impedance spectra (EIS) (Fig. S14). It is the tightened pore entrance (<0.4 nm), which is smaller than the sizes of solvent molecules and solvated Na^+^ (Fig. S15), that prevents the formation of an undesired IEDL as well as an SEI inside the nanopores.

### Unraveling the chemistry of the sodium clustering process

**Figure 2.**
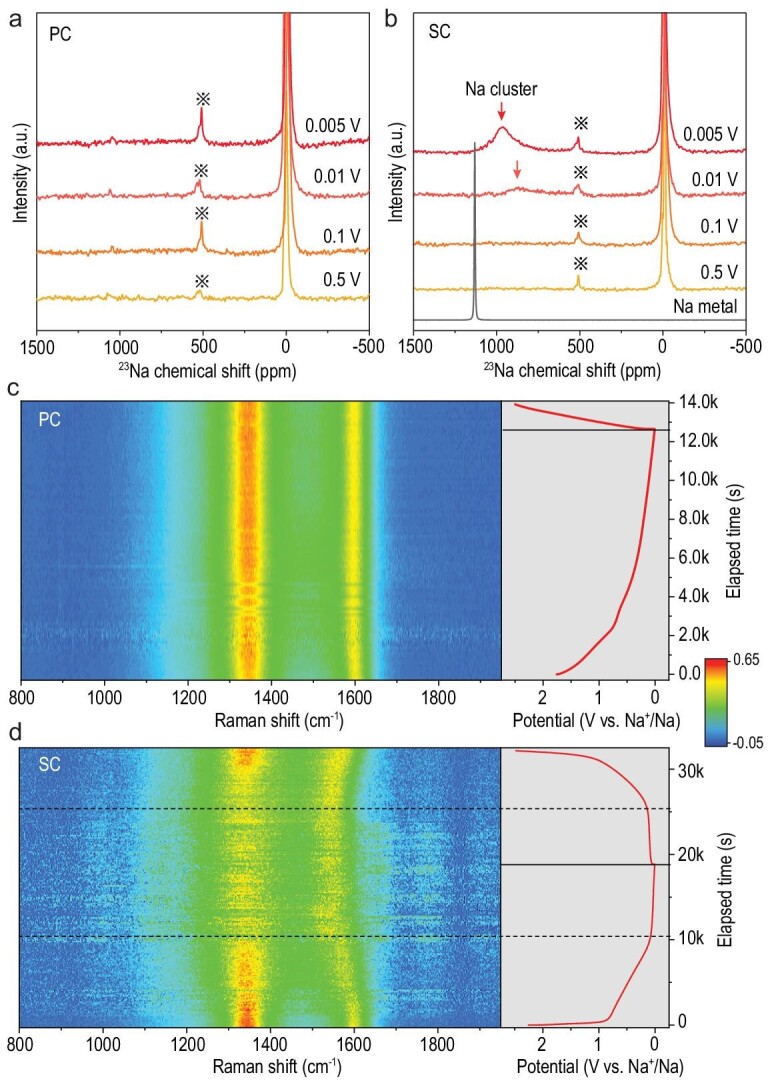
Characterizing the sodium clustering chemistry. (a and b) ^23^Na MAS ssNMR spectra of (a) PC and (b) SC anodes at various states of charge in the first cycle. The spinning sideband is labeled with an asterisk (*). (c and d) *Operando* Raman spectra of (c) PC and (d) SC anodes during the first charge/discharge at a current density of 50 mA g^–1^.

To reveal the LPP-related electrode chemistry, we used *ex-situ*^23^Na magic-angle-spinning (MAS) solid-state nuclear magnetic resonance (ssNMR) and *operando* Raman spectroscopy to probe the changes of the chemical states of both sodium and carbon, along with their interactions at different states of charge (Fig. 2; Figs S16 and S17). For the SC anode, only a sharp peak at around 0 ppm was observed in the ssNMR spectrum in the sloping region, which is ascribed to the formation of diamagnetic sodium ions in the bulk electrode and the SEI layer [18]. In the Raman spectrum, there is a prominent and reversible red-shift of the G-band from 1599 to 1550 cm^–1^, which is related to the electron–phonon coupling induced by electrons occupying the Π^*^ anti-bonding band of graphene nanosheets [34,35], indicating that the negative charges gradually transfer to the graphene nanosheets [36,37] and an ionic Na-C interaction is generated. In the plateau region, there is a peak centered at ∼960 ppm in the ssNMR spectrum arising from the Knight shift [38], related to the formation of quasi-metallic sodium clusters [18]. In addition, the G-band of SC remains almost constant, which confirms no electron occupation of the Π^*^ anti-bonding band of graphene nanosheets and that electrons are gradually transferred to sodium ions. Thus, a more metallic Na-Na interaction is generated with sodium ions as the electron acceptor, and the charge on the sodium ions is gradually decreased (Na^+^ → Na^δ^, δ < 1). The decreased charge repulsion causes most sodium ions to be stored in a clustered state with delocalized electrons. The ionic Na-C interaction in the sloping region is shown to be the prerequisite for the quasi-metallic Na-Na interaction in the plateau due to the insertion of abundant sodium ions into the nanopores.

For PC, there is neither a Knight shift in the ssNMR nor a G-band shift in the Raman spectra, suggesting capacitive sodium ion storage. Moreover, the relative intensity of the D-band remained almost constant for PC while there is a reversible change in intensity for SC, implying the critical role of defects in facilitating Na-C and Na-Na interactions in the SC anode, and highlighting the effectiveness of decreasing the PED to protect the defects and enable sodium clustering.

### Theoretical prediction for designing SC anodes with extended LPPs

**Figure 3.**
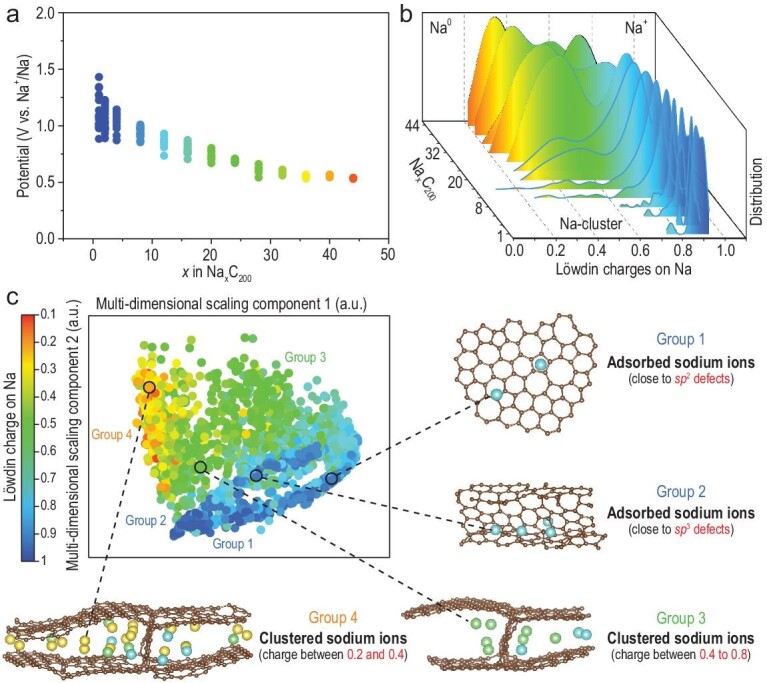
Theoretical insight into the dynamic sodium clustering process inside an SC anode. (a) Computed discharge potential curves consisting of discontinuous potential points, with a certain number of sodium atoms (taking Na_44_C_200_ as an example) inside the model carbon (the model is generated with 200 atoms per cell and the mass density is 1.153 g cm^–3^). (b) Löwdin charges (as computed using LOBSTER) for Na atoms with increasing numbers inserted from Na_4_C_200_ to Na_44_C_200_, drawn as kernel density estimated from the smoothed histograms. (c) Analysis of the local environment of stored sodium in SC using a smooth overlap of atomic positions kernel, as a structural similarity initially used for Gaussian approximation potential fitting. The map is obtained by multidimensional scaling based on the structural distances (representing the (dis)similarity). The most similar points are aggregated together with similar colors.

Theoretical simulations were conducted to provide atomistic insight into the dynamic sodium clustering process inside the SC anode, and predict how to design SCs for clustering more sodium ions to extend the LPP. An amorphous structural model for SCs with tunable nanopores was introduced, consisting of curved and defective graphene nanosheets (Fig. 3; Figs S18–S23). Based on this model, sodium ions were gradually inserted to simulate the dynamic sodium clustering process in an SC anode, temporarily ignoring any impact from the interfacial electrochemistry. The theoretically predicted discharge potential curve shows some similarities to the experimental results (Fig. 3a). During the initial stage of sodium insertion, there is a pronounced difference in maximum potentials, resembling the high-potential slope of an SC anode. Gradually increasing the number of sodium ions inserted led to similar maximum potentials, almost representing a plateau at low potentials.

The charge values at all accumulated sodium sites were collected during gradual sodium insertion (Fig. 3b). In the initial stage, when the number of inserted sodium ions (*x*) is below eight, the electrons of sodium are all localized and the charge number is ∼1, as for ideal sodium ions. With higher numbers of sodium ions inserted (*x* > 8), the electrons are gradually delocalized and the charge number is 0.2–0.6, implying partially ionic sodium, which is consistent with the quasi-metallic properties of sodium clusters certified by ^23^Na ssNMR.

To investigate the preferential sites for sodium clustering, a smooth overlap of atomic positions kernel was introduced [39,40]. The aim was to reveal the structural and chemical origins of sodium atoms with different charges, and generate a panoramic animation that depicts the evolution of the surrounding carbon environment during the gradual sodium clustering (Figs 3c and S21). Four distinct sodium groups were classified according to the different Löwdin charges and carbon environments. Group 1 is adsorbed sodium ions with mostly localized electrons and positive charges (higher than 0.8), which relates to preferential sodium storage close to the graphene sheets with many *sp*^2^ defects in the high-potential slope region. Group 2 has similar charge states but is in a different local carbon environment. In group 2, sodium ions are close to graphene sheets with *sp*^3^ defects, which typically link two adjacent graphene sheets. Group 3 is clustered sodium ions with partially delocalized electrons and charges lower than 1 (mostly between 0.4 and 0.8), which are surrounded by some aggregated sodium ions and which remain away from the graphene sheets. Group 3 only appears after the adsorbed sodium ions (Groups 1 and 2). Group 4 represents clustered sodium ions with more delocalized electrons whose charge number is close to a quasi-metallic sodium atom (between 0.2 and 0.4). In Group 4, sodium atoms are further away from the graphene sheets and are surrounded by many other clustered sodium ions. It is interesting to find that during sodium clustering, the different sodium states follow a strict sequential order, namely from sodium ions to sodium clusters, which corresponds to the different interactions between sodium and carbon, and is consistent with previous experimental results (Fig. 2). Therefore, in order to cluster more sodium ions to extend the LPP, PCs with a larger SSA are required to initially adsorb more sodium ions, theoretically guiding the experimental design of SC anodes with longer LPPs.

### Designing SC anodes with extended LPPs

**Figure 4.**
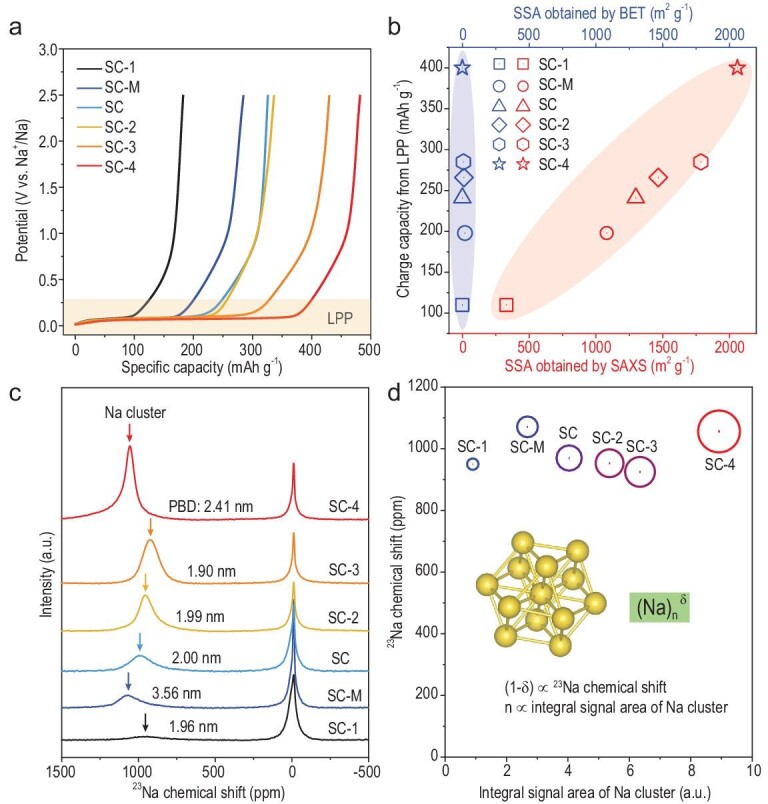
Correlation between the pore parameters and physicochemical properties of sodium clusters. (a) First-cycle charge curves of different SCs at a current density of 50 mA g^–1^. (b) Charge capacity from the low-potential plateau versus SSA obtained by SAXS and N_2_ adsorption for the SC anodes. (c) *Ex-situ*^23^Na ssNMR spectra of different SCs at 0.005 V for the first discharge. The PBDs of SCs are marked next to the peak of the Na cluster. (d) ^23^Na chemical shift versus integral signal area of Na clusters for different SCs. The radius of the round circle is proportional to the charge capacity from the low-potential plateau. Inset: schematic of an Na cluster, with (Na)_n_^δ^ as its simplified chemical formula, where n is the number of clustered sodium ions in a cluster and (1-δ) is the average transferred charge per clustered sodium atom.

With the above theoretical insight, we selected several commercial PCs (mostly microporous carbons together with a mesoporous carbon, denoted as PC-M) with different SSAs (based on N_2_ adsorption) as precursors, and used controlled CVD to produce target SCs until their nanopores could not be detected by N_2_. The SCs were denoted as SC-X (starting from microporous carbons, X represents the sequential order of increasing SSA) and SC-M (derived from PC-M). The detailed structure comparisons for PCs and the resultant SCs are seen in Figs S24–S26. The PEDs of all SCs were reduced to <0.4 nm, which was proven by the almost undetectable N_2_ adsorption (Fig. S24). The SSA of the SCs, calculated by SAXS, varied from 330 to 2059 m^2^ g^–1^, and the PBD of the SCs, calculated by SAXS, varied from 1.90 to 2.41 nm (Table S2). As expected, all the SCs had apparent LPPs when used as anodes in SIBs (Fig. 4a; Figs S27 and S28; Table S3) and their plateau capacities were approximately linearly correlated with the SAXS-based SSA (Fig. 4b). Based on this trend, we selected a commercial PC (YP-80 from Kuraray) with an ultra-high SSA (2538 m^2^ g^–1^ based on SAXS and 2179.4 m^2^ g^–1^ based on nitrogen adsorption) as the precursor, and the resultant SC (SC-4) achieved a record-high plateau capacity over 400 mAh g^–1^ and a total reversible capacity of 482 mAh g^–1^. Though various types of PCs were used to prepare SCs, they had similar ICEs (∼80%) and could be further improved.

Since the LPP originates from the sodium clustering process, the plateau capacity of SCs should be determined by the physicochemical properties of these clusters, namely the product of average transferred charge per clustered sodium atom and the number of clustered sodium ions in a unit mass of the SC, which can be obtained from ^23^Na ssNMR results, as shown in Fig. 4c and d. The transferred charge per sodium ion can be estimated from the chemical shifts, which are dominated by Knight shifts arising from the interaction of nuclear spins with unpaired Na 2s electrons located at the Fermi level of the conduction band [18,38]. The bigger the PBD of the SC, the larger the chemical shift of the sodium clusters inside, corresponding to a higher average transferred charge per sodium ion (Fig. 4c). When the average PBD exceeds 2.0 nm, taking SC-4 (∼2.41 nm) as an example, the chemical shift (1055 ppm) approaches 1130 ppm, which is characteristic of sodium metal [14,41]. When the average PBD increases to 3.56 nm, the so-called SC-M derived from a mesoporous carbon (PC-M) (Figs S29–S32), the chemical shift is still close to that of SC-4, only slightly different from 1130 ppm (Fig. 4c). Therefore, there is no need to further increase the PBD when it exceeds 2.0 nm, due to the negligible increase of average transferred charge per sodium ion. The integrated signal area in the ssNMR spectrum is linearly proportional to the number of probed nuclei (spins) [42], and thus the integrated signal area of sodium clusters should be proportional to the total number of clustered sodium ions in a unit mass of the SC. With the increased SSAs of SCs, it is apparent that the obtained integrated signal area of sodium clusters inside SCs is bigger, along with more clustered sodium ions and larger plateau capacity. Although SC-M has the largest PBD, it still delivers a low plateau capacity, since its relatively low SSA leads to a small number of clustered sodium ions inside.

### SC anodes with reversible and high-rate LPPs

**Figure 5.**
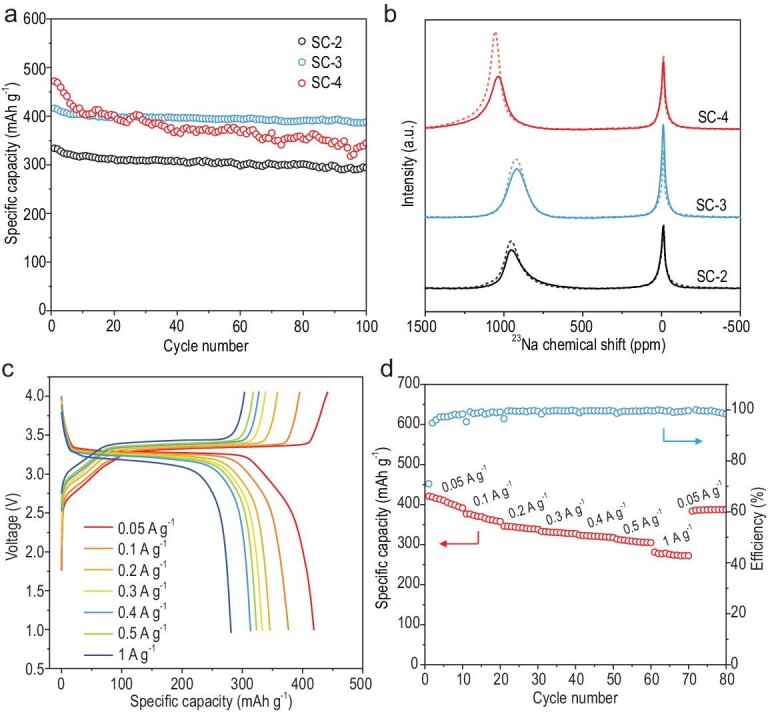
Change of sodium clusters in SC anodes during cycling, and full-cell performance. (a) Cycling performance of SC-2, SC-3 and SC-4 at 50 mA g^–1^. (b) *Ex-situ*^23^Na 55 kHz ssNMR spectra of SCs at 0.005 V for the 10th discharge at 50 mA g^–1^ (solid curves). The dashed curves are the corresponding ssNMR spectra of SCs at 0.005 V for the first discharge. (c) Charge/discharge curves and (d) rate performance of a Na_3_V_2_(PO_4_)_3_//SC-3 full cell from 0.05 A g^–1^ to 1 A g^–1^.

Electrochemical cycling at a relatively low current density was conducted to evaluate the reversibility of the formation of different sodium clusters (Fig. 5a). SC-3 had the best cycling stability, with a capacity retention of 93% after 100 cycles, while SC-4 had the highest plateau capacity but a capacity retention of only 73%. SC-3 had a high reversible capacity of around 390 mAh g^–1^ after 100 cycles and stable cycling at higher current densities (Figs S33 and S34). ^23^Na ssNMR spectra at a discharge potential of 0.005 V during the 10th cycle were collected to clarify the physicochemical property change of sodium clusters after cycling (Fig. 5b). For SC-2 and SC-3, with PBDs <2.0 nm, the ^23^Na chemical shifts of the sodium clusters remained almost unchanged. The slightly decreased integrated peak areas may be partially ascribed to the electrochemical polarization during prolonged cycling, which could be resolved by further electrolyte modification. In sharp contrast, for SC-4, with a larger PBD of 2.41 nm, an obvious ^23^Na chemical shift from 1055 to 1037 ppm was seen, together with substantially decreased integrated peak areas, implying that sodium clusters with a higher metallicity have inferior reversibility during cycling. This highlights the importance of having a relatively small PBD when designing practical SC anodes. Additionally, the tightened PEDs of SCs may limit the entrance of electrolyte solvents and thus inhibit their reaction with the highly reactive sodium clusters (Fig. S15), contributing to the reversibility of the sodium clusters and resulting LPPs. In brief, the design of practical SCs for high-energy SIBs with reversibly extensible LPPs requires a small PED (<0.4 nm), and a high SSA, with the upper PBD limit of ∼2.0 nm.

Full cells with a Na_3_V_2_(PO_4_)_3_ cathode and SC-3 anode were assembled and had a record-high reversible capacity of 421 mAh g^–1^ (based on the anode mass) and energy density of 171 Wh kg^–1^ (based on cathode and anode mass), with an average voltage of 3.3 V at 0.05 A g^–1^ (Fig. 5c). A superior rate capability was also obtained, with reversible capacities of 314 and 281 mAh g^–1^ at 0.5 A g^–1^ and 1 A g^–1^, respectively (Fig. 5d), outperforming most reported carbon anodes in SIBs [43–46].

## CONCLUSION

We propose sieving carbons as high-energy anodes for practical SIBs, featuring highly developed nanopores coupled with a tightened pore entrance. Using sieving carbons as anodes not only produces a critical LPP similar to that of the graphite anode for LIBs, but also reversibly extends the LPP to deliver even higher capacities and better rate capability. The limited PED (<0.4 nm) of sieving carbons is proven to be a prerequisite for sieving the solvated sodium ions and facilitating sodium clustering inside the nanopores to produce the LPP. With spectroscopic and theoretical insight, we show that the number of clustered sodium atoms increases linearly with the SSA of sieving carbons, directly contributing to the extension of the LPP and a record-high plateau capacity of 400 mAh g^–1^. Furthermore, controlling the metallicity of sodium clusters with an upper-limit PBD (<2.0 nm) guarantees the reversibility of the LPP of sieving carbon anodes. The current approach to producing sieving carbons is simple and has the potential to be scalable with regard to upgrading most commercial PCs, and promises to accelerate the large-scale implementation of SIBs.

## METHODS

### Material synthesis

PCs were purchased from Kuraray (Japan). Mesoporous carbon was purchased from Nanjing Ji Cang Nano Technology Co., Ltd (China). Sieving carbons were prepared from these carbons by heat treatment from 25°C to 900°C in a horizontal tube furnace, with a heating rate of 5°C min^–1^ under an argon atmosphere (90 mL min^–1^), followed by CVD using methane as the precursor with a flow rate of 10 mL min^–1^ mixed with argon (vol% = 1:9). The mass of PC in the tube furnace was 0.5 g. To investigate the effect of PED on the electrochemical performance, one PC was treated with different deposition times (0 h, 0.5 h, 2 h and 5 h) to change the PED value, and these are denoted as PC, SC-0.5h, SC and SC-5h. Other PCs, including one mesoporous carbon, were treated until their nanopores could not be detected by N_2_; these were denoted as SC-X (X represents the sequential order of increasing SSA) and SC-M. The corresponding carbons treated with a deposition time of 0 h were denoted as PC-X and PC-M. The PyC was deposited using a methane flow rate of 50 mL min^–1^ and an argon flow rate of 50 mL min^–1^.

### Material characterization

Scanning electron microscopy (SEM) images were obtained using a Hitachi S4800 instrument. Transmission electron microscopy (TEM) images and selected area electron diffraction (SAED) were obtained using a JEOL JEM 2100F operating at 200 kV. N_2_ adsorption-desorption at 77 K and CO_2_ adsorption-desorption experiments at 273 K were conducted using a BELSORP MAX analyzer. The SSA and pore diameter distribution were analyzed with the Brunauer-Emmett-Teller (BET) method and the non-local density functional theory (NLDFT) method, respectively. X-ray diffraction (XRD) was carried out on a Rigaku D/Max 2500 PC diffractometer using CuKa radiation (λ = 1.54056 Å) and Raman spectra were recorded on MicroRaman system (LabRAM HR spectrometer, Horiba). The XPS measurements were performed using an ESCALAB Xi+ (Thermo Fisher Scientific) with a focused monochromatic Al X-ray source. SAXS was performed using a Xeuss 2.0 SAXS/WAXS System with a Cu X-ray source of 30 W (wavelength = 0.1542 nm).

### Electrochemical measurements

Cyclic voltammetry (CV) measurements were collected at a scanning rate of 0.1 mV s^–1^ in the potential range of 0.005–2.5 V vs. Na^+^/Na, and EIS were measured in the frequency range of 10 mHz–100 kHz with a PC signal amplitude of 10 mV. Both were tested on an electrochemical workstation (Eco Chemie Autolab). The galvanostatic charge/discharge measurements were on a Neware battery cycler (CT-4008T-5V20mA-164, Shenzhen, China).

## Supplementary Material

nwac084_Supplemental_File
