# Supplementary material for: Sieving carbons promise practical anodes with extensible low-potential plateaus for sodium batteries
Source: Natl Sci Rev. 2022 May 5;9(8):nwac084. doi: 10.1093/nsr/nwac084 (PMC9385462; doi:10.1093/nsr/nwac084)
Supplement: nwac084_Supplemental_File [file nwac084_supplemental_file.docx]

**Sieving carbons promise practical anodes with extensible low-potential plateaus for sodium batteries**

Qi Li^1,2,4,†^, Xiangsi Liu^3,†^, Ying Tao^1,4,†^, Jianxing Huang^3^, Jun Zhang^2,1,4,*^, Chunpeng Yang^1,4^, Yibo Zhang^1,4^, Siwei Zhang^5^, Yiran Jia^1,4^, Qiaowei Lin^5^, Yuxuan Xiang^3^, Jun Cheng^3^, Wei Lv^5^, Feiyu Kang^5^, Yong Yang^3,*^, Quan-Hong Yang^1,2,4,*^

^1^Nanoyang Group, State Key Laboratory of Chemical Engineering, School of Chemical Engineering and Technology, Tianjin University, Tianjin 300072, China.

^2^Nanoyang Group, Joint School of National University of Singapore and Tianjin University, International Campus of Tianjin University, Binhai New City, Fuzhou 350207, China.

^3^State Key Laboratory of Physical Chemistry of Solid Surfaces, Collaborative Innovation Center of Chemistry for Energy Materials and Department of Chemistry, College of Chemistry and Chemical Engineering, Xiamen University, Xiamen 361005, China.

^4^Haihe Laboratory of Sustainable Chemical Transformations, Tianjin 300192, China.

^5^Shenzhen Key Laboratory for Graphene-based Materials, Engineering Laboratory for Functionalized Carbon Materials, Tsinghua Shenzhen International Graduate School, Tsinghua University, Shenzhen 518055, China.

***Corresponding authors**. E-mails: zhjun20@tju.edu.cn; yyang@xmu.edu.cn; [qhyangcn@tju.edu.cn](mailto:qhyangcn@tju.edu.cn)

^†^Equally contributed to this work.

**Supplementary methods**

**1. Battery assembly**

The electrochemical performance was tested in 2032-type coin cells at room temperature which were assembled in vacuum glove-box. For the preparation of the electrodes, the active materials such as porous carbons and sieving carbons, polyvinylidene fluoride (PVDF) and carbon black with mass ratios of 8:1:1 were mixed in N-methyl-2-pyrrolidone (NMP) and coated on a Cu foil. The mass loading of the electrodes was ~1.2 mg cm^-1^. Sodium foil was used as the counter/reference electrode in the SIBs. The electrolyte was composed of 1M NaClO_4_, (Aladdin, China) solution in a mixture of ethylene carbonate (EC) and diethyl carbonate (DEC) (1:1 in volume). A mat of glass fibers (19 mm in diameter, GF/A Whatman) was used as the separator. Sodium-ion full cells were constructed using Na_3_V_2_(PO4)_3_ as the cathode material and SC-3 as the anode material. The anode/cathode mass ratio was 1:5.2. The full cells were charged and discharged in the voltage range of 1-4.05 V.

**2. Small angle X-ray scattering (SAXS)**

SAXS was performed with a Cu X-ray source of 30 W (wave length of 0.1542 nm) and an X-ray spot size of 0.8 mm × 0.8 mm. The scattering vector (*Q*) ranges were adjusted using a sample-camera distance of 0.55 m for 1800 s to cover a broad angular range using the virtual detector mode of three combined images, and the corresponding scattering vector *Q* value was 0.01–1.1 Å^-1^. The two-dimensional scattering data were transformed to one-dimension data curves with Foxtrot 3.2.4 software. The measured intensity was corrected for absolute intensity using glassy carbon as a standard sample and calibrated to exclude the influence of all related factors including sample thickness, air and sample holder scattering etc.^[[1](#_ENREF_1)]^ The SAXS data were analysed using the SasView 4.2.2 software.

**3. Solid electrolyte interphase (SEI) characterization**

The batteries were disassembled in a glove box after de-sodiation in the 10^th^ cycle, and the electrodes were rinsed several times with DEC solvent. After drying, the electrodes were packed in a vacuum chamber and transferred into the XPS chamber. The obtained electrodes were ion-etched using 3000 e V Ar ions over 2000 μm^2^ for 20 min.

*Ex-situ* SAXS was performed using a Xeuss 2.0 SAXS/WAXS System and the testing method is described in detail in **Small angle X-ray scattering (SAXS)**. The batteries were disassembled in a glove box after de-sodiation in the 5^th^ cycle, and the electrodes were rinsed several times with DEC solvent. After drying, the electrodes were collected from the Cu foil and transferred into the sample holder with a plastic film on the top. To avoid exposure to air, the holder was packed in an Al pouch until the holder was removed and transferred to the SAXS chamber prior to testing.

**4. *Operando* Raman**

*Operando* Raman spectra were obtained on a MicroRaman system (LabRAM HR spectrometer, Horiba) with an argon ion laser (532 nm). The electrodes were prepared by mixing the active materials, PTFE, and carbon black in mass ratios of 90:5:5 in deionized water and rolled into a thin film and pressed onto a stainless-steel mesh. A quartz window on the top of cell was fabricated for the laser to pass through and each spectrum was acquired for 60 s.

**5. *Ex-situ* ^23^Na solid-state nuclear magnetic resonance (ssNMR)**

The ^23^Na ssNMR experiments with/without magic-angle-spinning (MAS) were performed on a Bruker AVANCE III 400 MHz spectrometer with a 1.3 mm double-resonance HX probe. A ^23^Na Hahn-echo pulse sequence (90°- τ - 180°- τ) experiment was performed with a 90° pulse length of 2 μs (25W) and a delay time of 0.05 s to ensure quantitative analysis. The ^23^Na chemical shifts were calibrated using a 1 M NaCl aqueous solution (0 ppm) as external reference. The rotors spun at 50 or 55 kHz to distinguish different resonance signals and the rotors were tested under static conditions for quantitative analysis. All the *ex-situ* ssNMR spectra were normalized with respect to the mass of sample in the rotors and number of scans. For ssNMR measurements, the batteries were disassembled in a glove box after the 1^st^ and 10^th^ sodiations at a rate of 50 mA g^-1^ until a cutoff potential of 0.005 V was reached. The electrodes were collected from the Cu foil and transferred into the 1.3 mm rotor without rinsing and sealed with a Vespel cap in a short time so that exposure to air or moisture was avoided.

**6. Theoretical simulations**

The non-graphitic model carbon generated by machine learning potential methods from previous reports was used in this work^[[2](#_ENREF_2)]^. Three model carbons were generated with 200 atoms per cell only with different mass densities of 0.699, 1.153 and 1.491 g cm^-3^, while keeping the shortest-path ring statistics fixed (13% 5-/7-membered rings, 6% *sp*^3^ carbons and 94% *sp*^2^ carbons). As a result, their respective average PBDs were 0.7, 0.8 and 0.9. We simulated the insertion of sodium atoms in the model carbons with DFT computations and the computed cell potential as a function of sodium filling was explored.

In the computed discharge potential profiles, the potential *U* was obtained as

$$\begin{aligned} U=-\frac{1}{x}\left[ E_{DFT}\left( M_{x}C_{y} \right)-xE_{DFT}\left( M \right)-yE_{DFT}\left( C \right) \right]\#\left( 1 \right) \end{aligned}$$

where *E* is the energy for a given component obtained from DFT computations at 0 K, *M* is sodium atom, *C* is carbon atom, *x* is the number of inserted sodium atoms, *y* is the number of carbon atoms in the model, and van der Waals (vdW) interactions were described using the optB88-vdW functional^[[3](#_ENREF_3)]^. Specifically, the projector augmented-wave (PAW) method as implemented in VASP 5.4.4 was chosen. In this work, rather than defining staging compounds formed in the crystalline materials, we sampled a large number of configurations to sample possible electrochemical active sites. As the number of scatter points decreased, we started with 20 independent computations at low sodium concentrations and reduced the number of sampled cells to 5 at larger sodium concentration.

Initially, the atomic coordinates in all structures were relaxed using DFT until residual forces fell below 0.01 eV Å^-1^. In these computations, reciprocal space was sampled at the Γ point, and an electronic smearing of σ = 0.2 eV and a cut-off energy of 500 eV were used; cell shape and volume were kept fixed. For the optimized structures, more accurate single-point computations were performed, using a grid of 1 × 1 × 1 *k*-points, σ = 0.1 eV, and a cut-off energy of 500 eV. Previous convergence tests confirmed that 1 × 1 × 1 *k*-points is enough to ensure that the energy is converged to 1meV/atom.

The smooth overlap of atomic positions (SOAP) descriptor was used to identify the local environment of each sodium atom. We used a dimensionality reduction technique, namely principal component analysis (PCA), to reduce the descriptors to two dimensions. The data points were color-coded based on the DFT-computed Löwdin charges on each sodium atom, and aggregated according to the local environment. Therefore, the similarities of local structure were visualized to identify the relationships between the different local environments of sodium during the insertion process.

**Note S1. Models for SAXS data analysis.**

According to the method proposed by Damien Saurel, the semi-empirical Teubner-Strey model was used to analyze the porous structure^[[1](#_ENREF_1), [4](#_ENREF_4), [5](#_ENREF_5)]^:

$$\begin{aligned} I_{mp}=I_{0}\frac{1}{1+C_{1}Q^{2}+C_{2}Q^{4}}\#\left( 2 \right) \end{aligned}$$

where the parameters I_0_, C_1_ and C_2_ are defined as:

$$\begin{aligned} I_{0}=\frac{8\pi}{\rho_{s}}\phi\left( \Delta SLD \right)^{2}\frac{\xi^{3}}{\left( 1+\left( \frac{2\pi\xi}{d} \right)^{2} \right)^{2}}\#\left( 3 \right) \end{aligned}$$

$$\begin{aligned} C_{1}=\frac{-2\xi^{2}\left( \frac{2\pi\xi}{d} \right)^{2}+2\xi^{2}}{\left[ 1+\left( \frac{2\pi\xi}{d} \right)^{2} \right]^{2}}\#\left( 4 \right) \end{aligned}$$

$$\begin{aligned} C_{2}=\frac{\xi^{4}}{\left[ 1+\left( \frac{2\pi\xi}{d} \right)^{2} \right]^{2}}\#\left( 5 \right) \end{aligned}$$

where d is the average distance between two adjacent pores, ξ is a correlation length limiting the extension of the long-range order.

The average pore body diameter D can be calculated from the formula:

$$\begin{aligned} D=2\sqrt{5C_{1}}\#\left( 6 \right) \end{aligned}$$

The SSA is estimated by analogy with the Porod law^[[6](#_ENREF_6)]^:

$$\begin{aligned} S_{mp}=\frac{I^{0}}{C_{2}\left( \Delta SLD \right)^{2}}\#\left( 7 \right) \end{aligned}$$

where ΔSLD is the difference of scattering length density between the pores and the carbon matrix of the samples. The SLD value of the pores is zero because the pores are empty voids. The SLD of the carbon matrix can be calculated by SLD Calculator of the SasView 4.2.2 software using ρ_s_, where ρ_s_ is the structural density calculated by the equation:

$$\begin{aligned} {}_{s}=\rho_{g}\frac{d_{002}}{{d_{002}}^{g}}\left( \frac{d_{100}}{{d_{100}}^{g}} \right)^{2}\#\left( 8 \right) \end{aligned}$$

where ρ_g_ is the theoretical density of graphite (2.26 g cm^-3^). d_002_ and d_002_^g^ are the average interlayer spacings of the sample and graphite, d_100_ and d_100_^g^ are the repeat distances of the (100) lattice planes in the sample and in graphite. The specific values of d_002_ and d_100_ were obtained from X-ray diffraction (XRD) patterns.


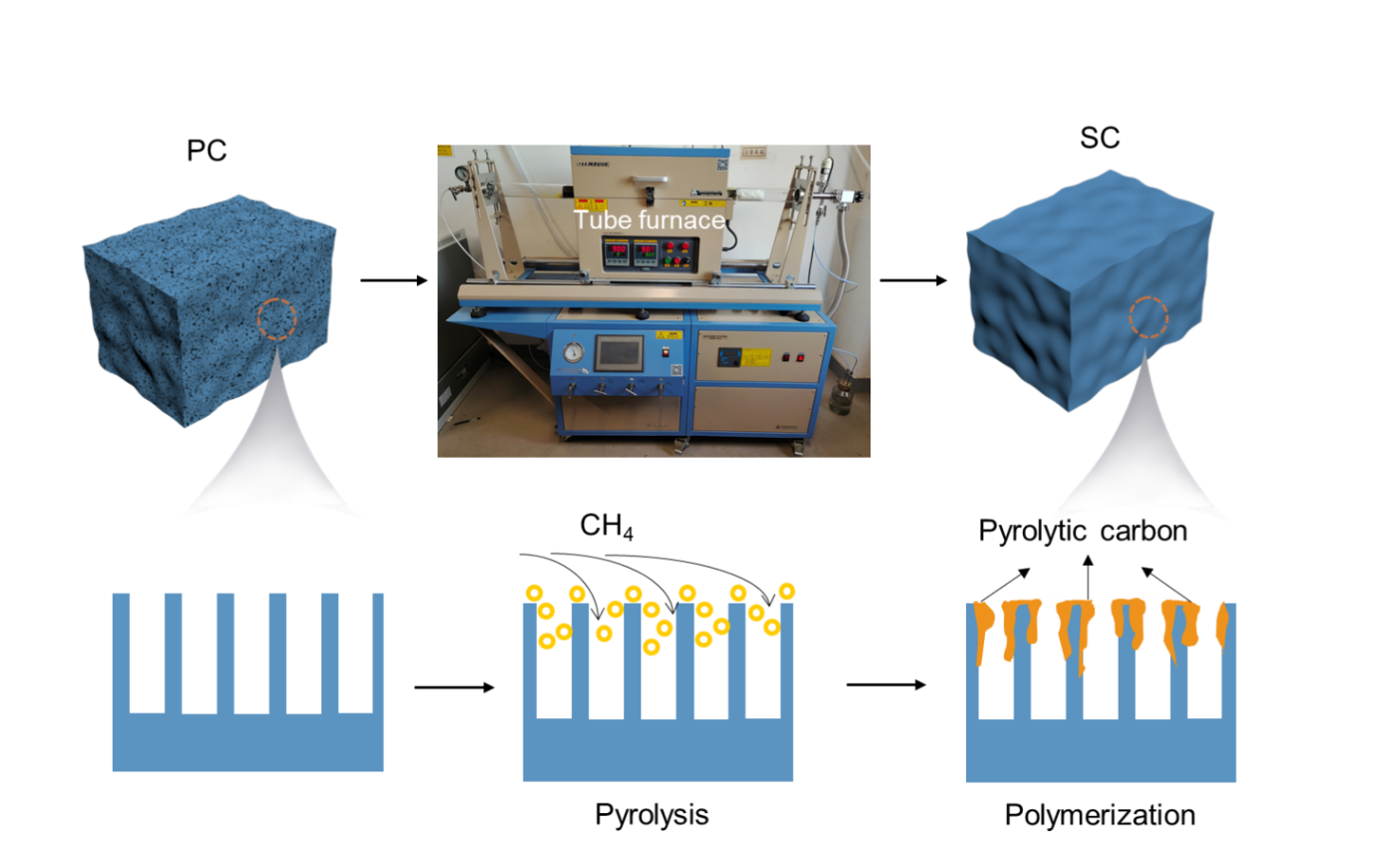


**Figure S1.** Schematic of the strategy to produce the SCs, namely CVD of methane on PCs using a laboratory tube furnace. The pyrolysis temperature here was set to 900 ℃ and the flow rate of methane was fixed at 10 mL min^-1^ mixed with argon (volume ratio=1:9), with only different deposition times.


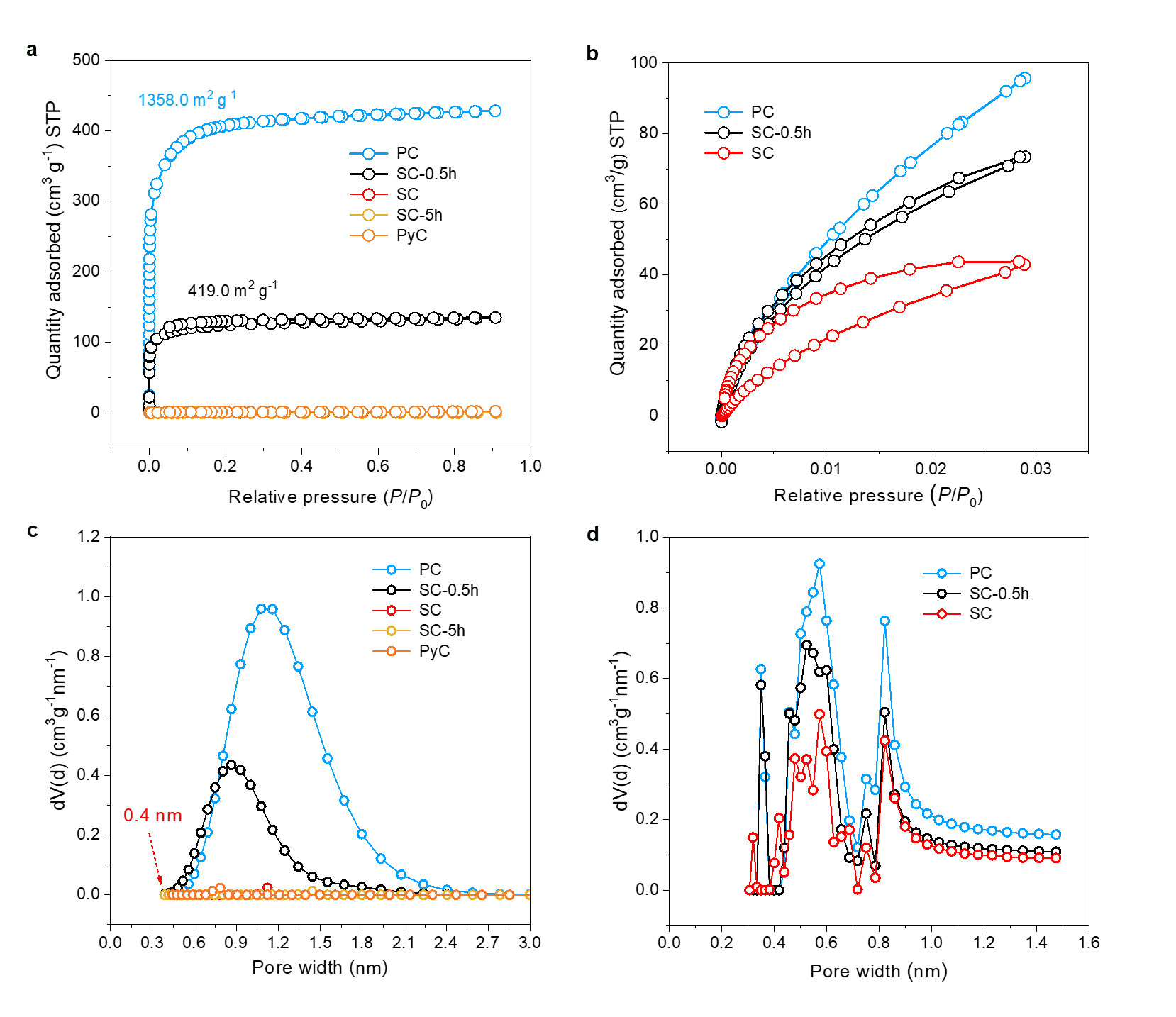


**Figure S2. Pore structure characterization of PC, SCs and PyC.** (a) N_2_ adsorption-desorption isotherms at 77 K and (b) CO_2_ adsorption-desorption isotherms at 273 K. (c, d) Pore diameter distributions based on (c) N_2_ adsorption-desorption isotherms and (d) CO_2_ adsorption-desorption isotherms.

In order to precisely control the pore entrance diameter (PED), both insufficient deposition and excessive plugging must be avoided. Therefore, the methane carbon source is controlled at a relatively low concentration at a moderate temperature. PC has an IUPAC Type I adsorption-desorption isotherm in both N_2_ (molecular dynamics diameter: ~0.37 nm) at 77 K and CO_2_ (molecular dynamics diameter: ~0.33 nm) at 273 K, indicating dominant micropores. As the deposition time increases, the SSA of the SCs tested by both N_2_ and CO_2_ gradually decreased with a pronounced closed hysteresis loop, ascribed to the gradually increasing difficulty of N_2_ and CO_2_ desorption caused by the lower PED^[[7](#_ENREF_7), [8](#_ENREF_8)]^. Specifically, pore diameter distributions obtained by N_2_ shows that all pores with a PED (pore width in Supplementary Fig. 2c) greater than 0.4 nm were not detected when the deposition time was increased to 1 h, which suggests that the PED of all the pores is gradually regulated to less than 0.4 nm. By extending the deposition time to 5 h, porosity in SC-5h can hardly be detected by both molecular probes, suggesting that the PED is smaller than 0.33 nm^[[9](#_ENREF_9)]^.


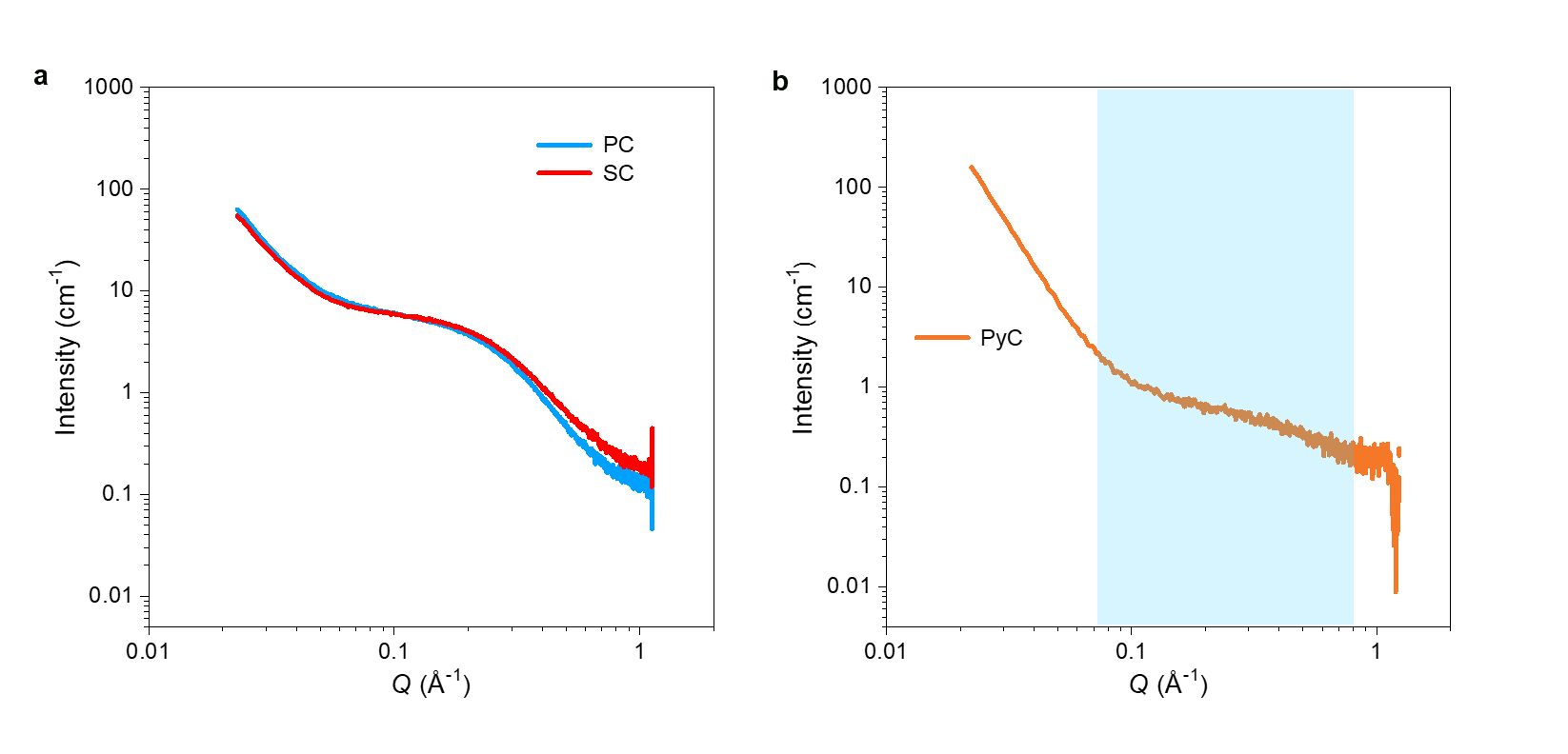


**Figure S3.** SAXS pattern of (a) PC and SC, and (b) PyC. The absence of microstructural features, namely a broad peak in the intermediate Q range (highlighted in light blue), suggests that the carbon produced by methane deposition is dense and non-porous.

**
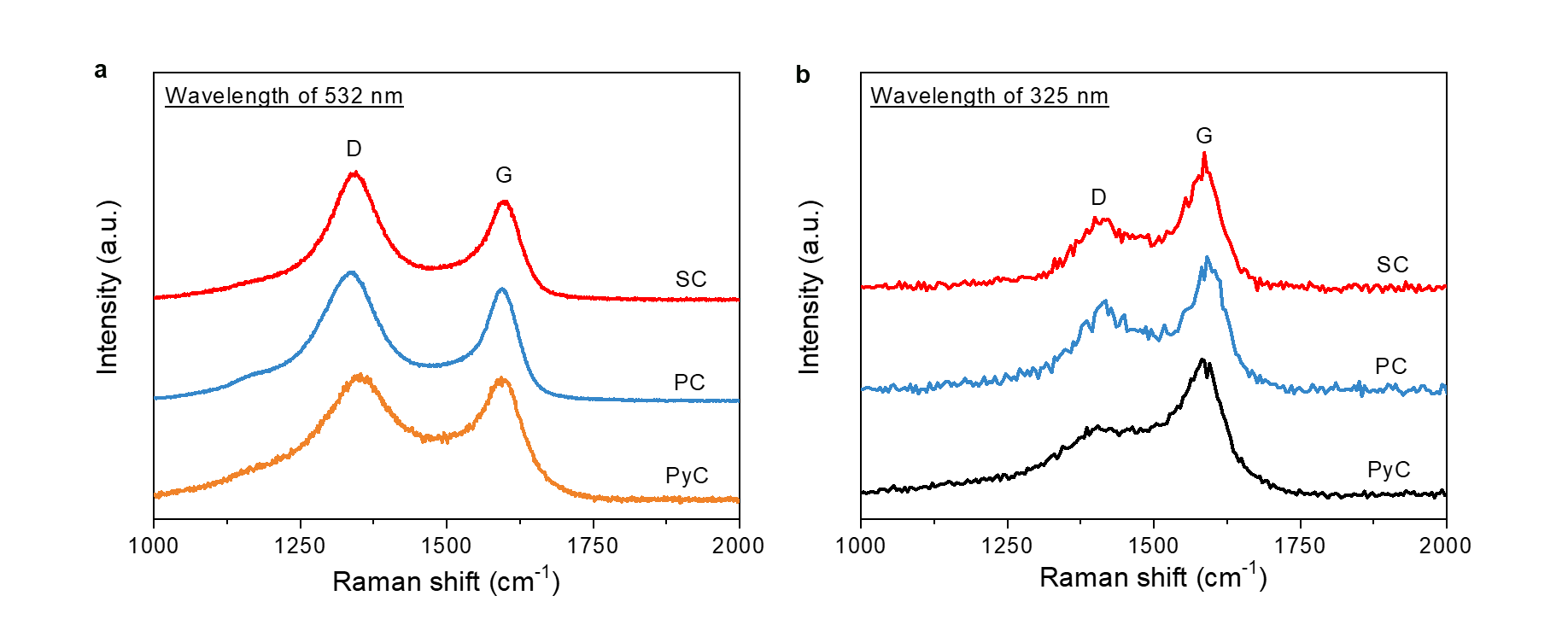
**

**Figure S4.** Raman spectra with wavelengths of (a) 532 nm and (b) 325 nm of PC, SC and PyC.


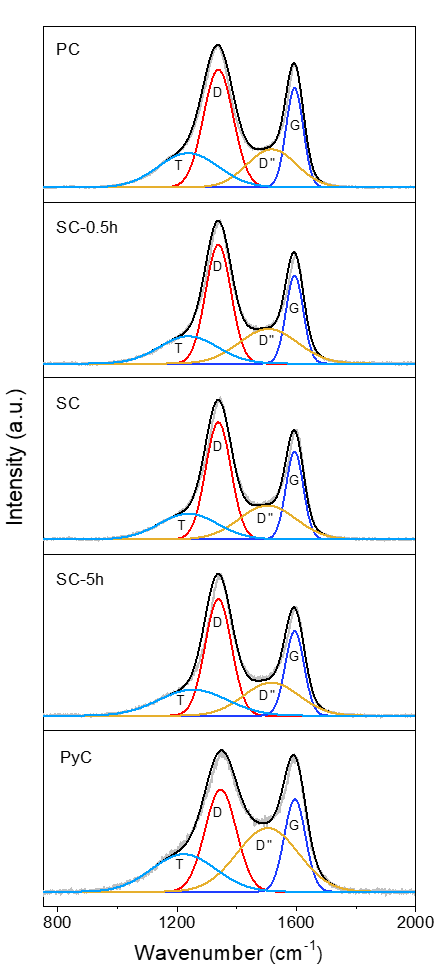


**Figure S5.** Fitted Raman spectra with wavelength of 532 nm of PC, SCs and PyC.

The Raman spectra have two typical characteristic peaks of the D-band at around 1340 cm^-1^ arising from *sp*^2^ carbon atoms in rings at edge planes and defects in graphene sheets, and a G-band at around 1590 cm^-1^ ascribed to the aromatic *sp*^2^ carbon atoms^[[10-12](#_ENREF_10)]^. The corresponding fitted Raman spectra consists of four components in the 800-2000 cm^-1^ range, expressed as T-, D-, D”-, and the G-band^[[13](#_ENREF_13)]^.

The integrated intensity ratio I_G_/I_D_ from the Raman spectra can be used to quantify the defect concentration and the corresponding average width of the graphitic micro-crystallites (L_a_), which can be calculated from the following formula^[[14](#_ENREF_14)]^.

$$\begin{aligned} L_{a}\left( \mathrm{nm} \right)=\left( 2.4\times{10}^{-10} \right){\lambda_{nm}}^{4}\left( \frac{I_{G}}{I_{D}} \right)\#\left( 9 \right) \end{aligned}$$

where λ_nm_ is the wavelength of the laser, I_D_ and I_G_ are the integrated intensities of the D- and G-bands, respectively.

Calculated from Raman spectra obtained using a wavelength of 532 nm, the L_a_ values of PyC and PC are 9.59 nm and 6.56 nm, respectively, which suggests larger graphene aromatic sheets in PyC than in PC. However, the L_a_ of SC is unexpectedly lower at 5.07 nm. We therefore deduce that PyC gradually is deposited on the pore walls near the pore entrance of PC in the form of smaller graphene aromatic sheets due to the confining effect of the nanopores (limited space). From Raman spectra with a wavelength of 325 nm, which has a lower optical penetration depth and mainly provides superficial information, the L_a_ values of PC (15.19 nm) and SC (15.74 nm) are similar but much lower than that of PyC (24.06 nm). The distinct superficial properties of the latter two samples imply that PyC is mainly confined to the nanopores rather than fully coated on the surface of the PC, which is consistent with previous studies^[[15](#_ENREF_15)]^. However, by increasing the deposition time to 5 h, L_a_ increases slightly to 5.28 nm for SC-5h, implying that excess PyC with larger graphene aromatic sheets is coated on the surface of PC.


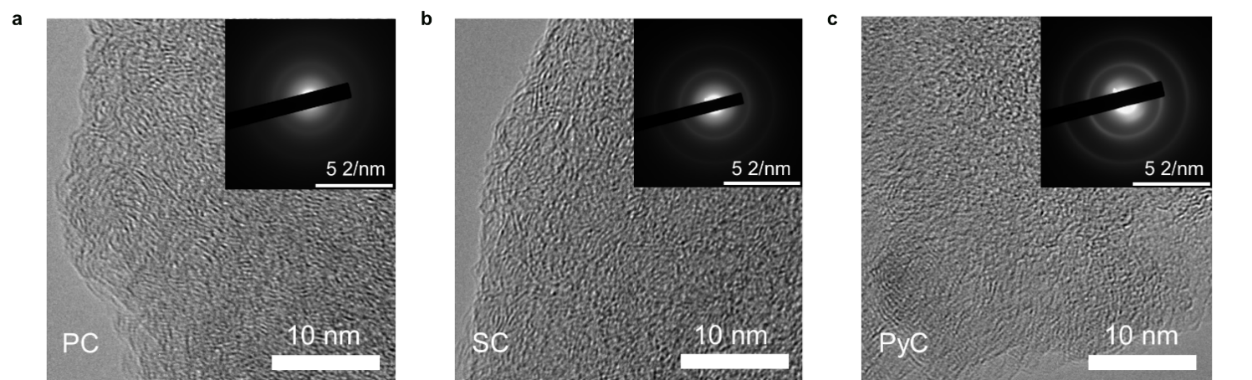


**Figure S6.** HRTEM and selected area electron diffraction patterns of (a) PC, (b) SC and (c) PyC.


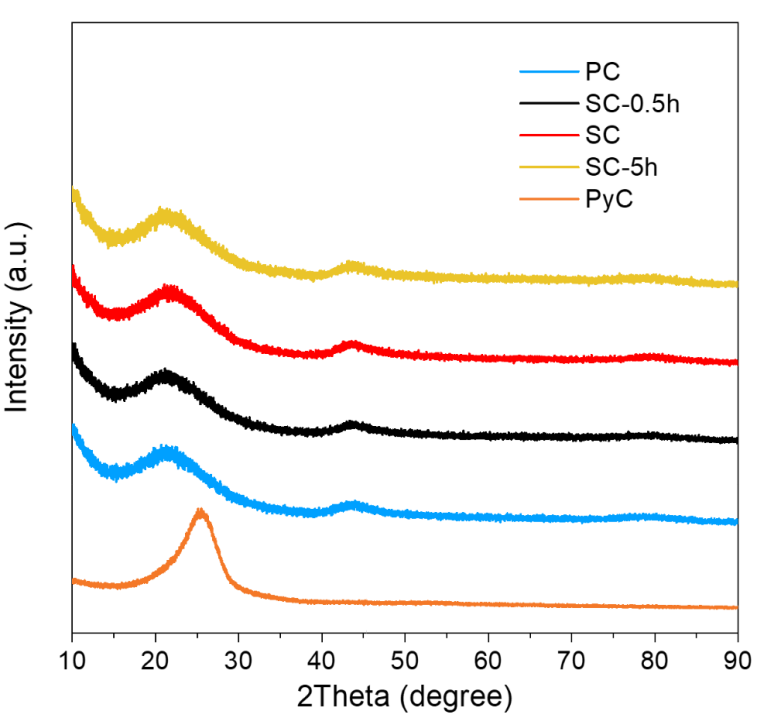


**Figure S7.** XRD patterns of PC, SCs and PyC.

The XRD patterns of PC, SCs and PyC all have two broad diffraction peaks at approximately 24^o^ and 46^o^ respectively corresponding to the (002) and (100) diffractions, which suggests a long-range disordered graphitic structure of the amorphous carbon. The interlayer spacings of PC, SCs and PyC were calculated using the Bragg equation and the average thickness (L_c_) of the graphitic crystallites were obtained using the Scherrer equation^[[16](#_ENREF_16)]^.


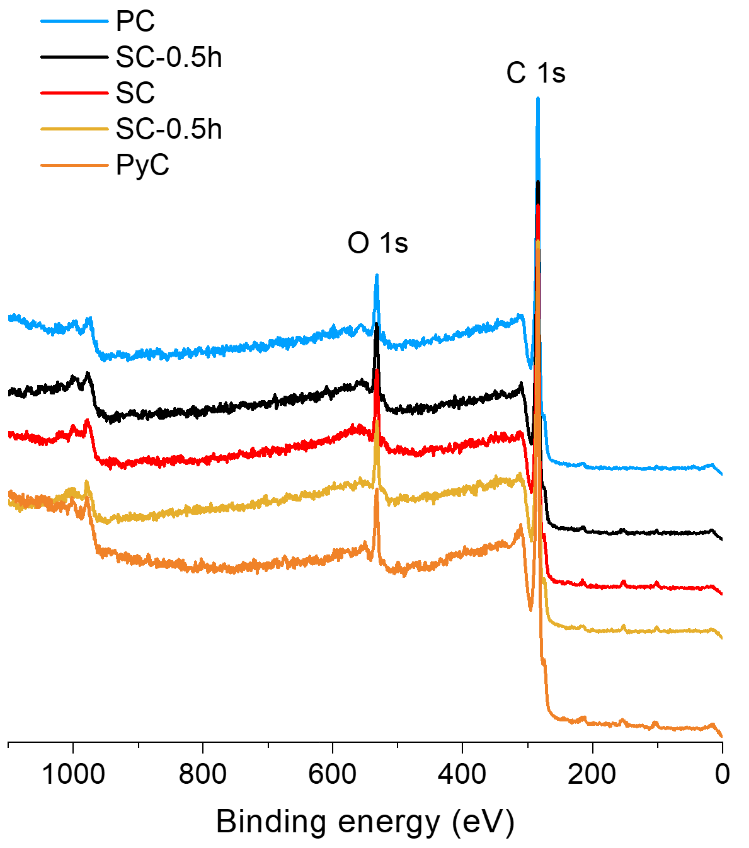


**Figure S8.** X-ray photo-electron spectroscopy (XPS) survey spectra of PC, SCs and PyC.


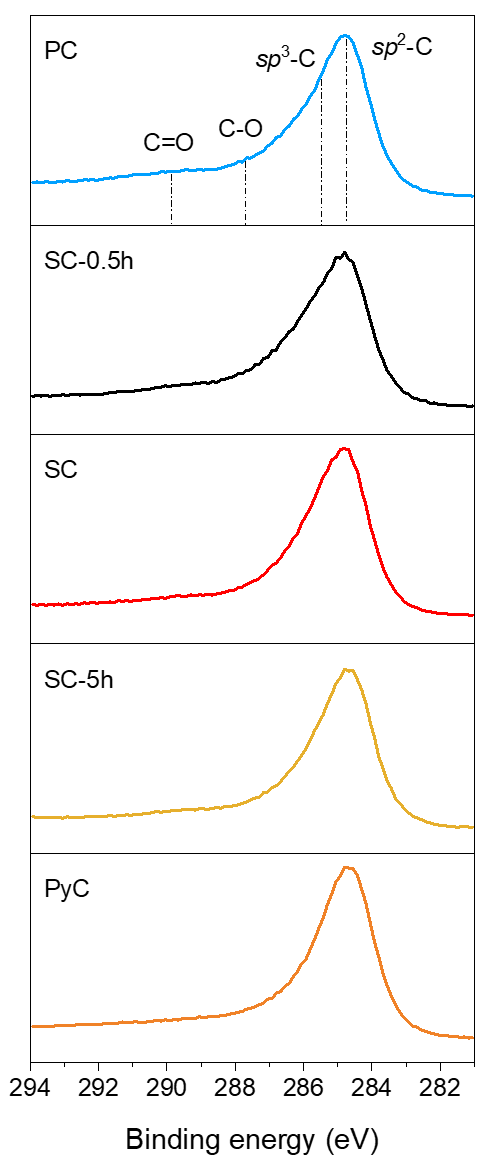


**Figure S9.** Fitted XPS C 1s spectra of PC, SCs and PyC.

Similar surface chemistry (C=O, C-O, *sp*^3^-C, *sp*^2^-C) is observed in the XPS C 1s spectra of SCs and PC, which proves the feasibility of the strategy to regulate the PED with negligible influence on other structural or chemical features.


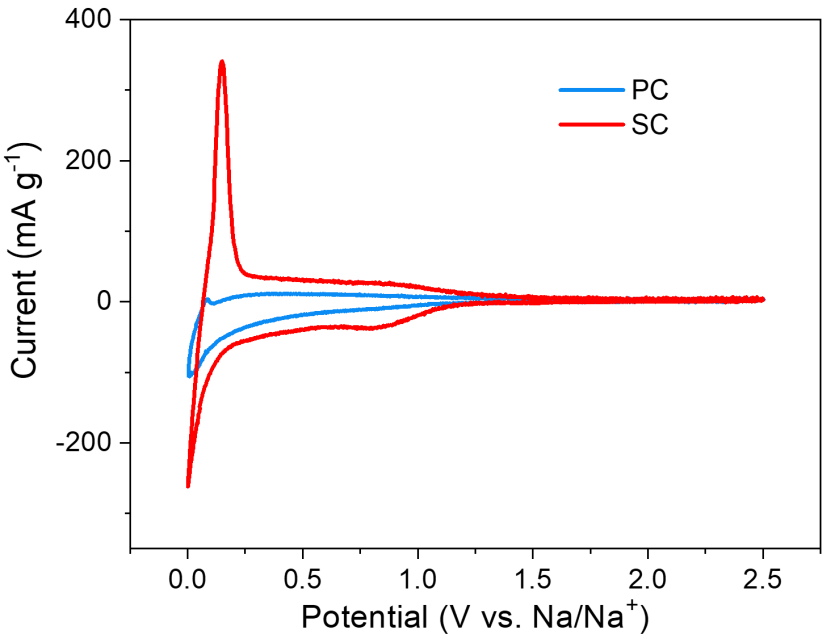


**Figure S10.** CV curves at a scan rate of 0.1 mV s^-1^ for the 1^st^ cycle of PC and SC anodes.

**
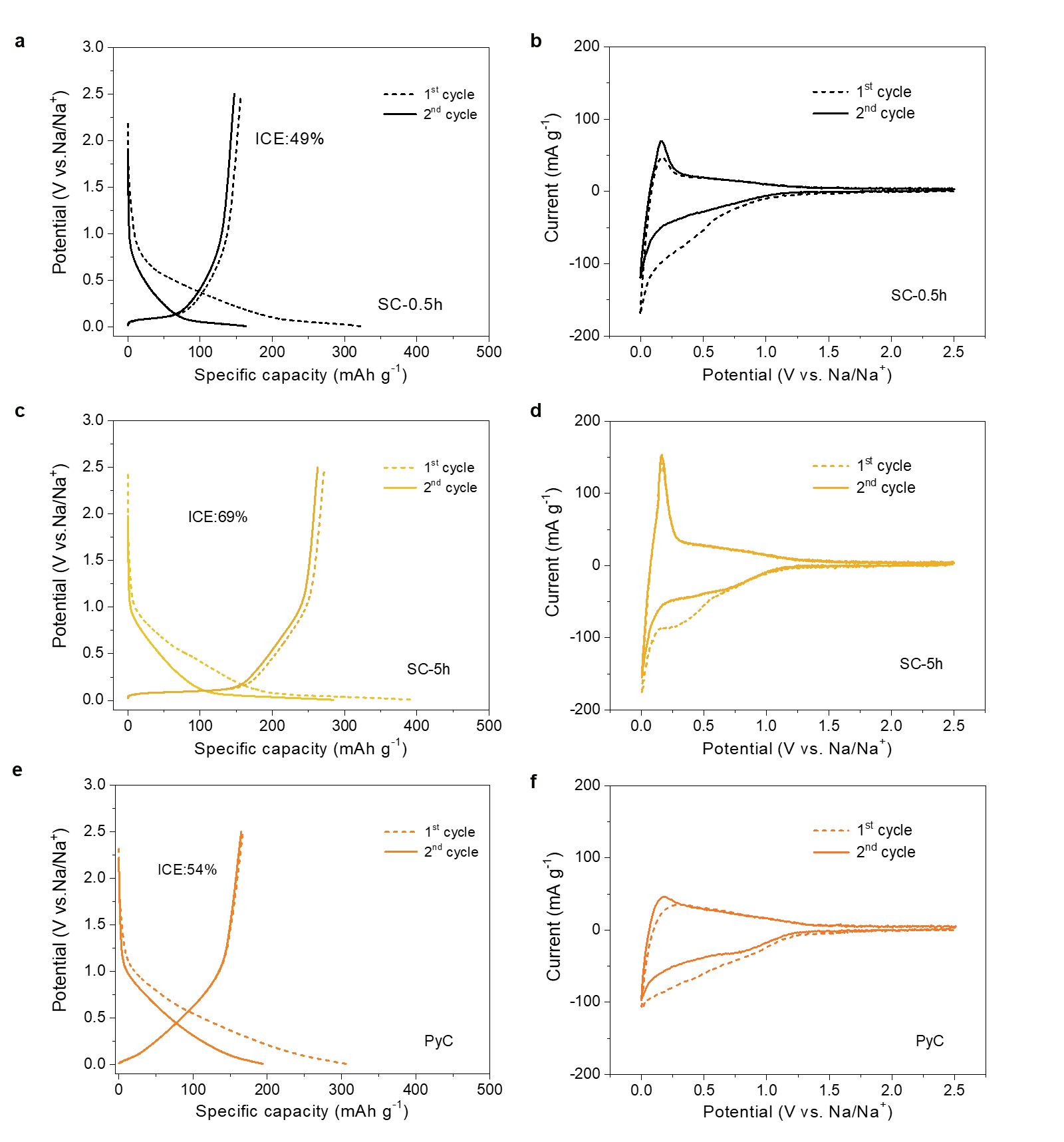
**

**Figure S11. Electrochemical performance of** **SC anodes and the PyC anode.** (a, c, e) Charge/discharge curves for the first two cycles at a current density of 50 mA g^-1^ and (b, d, f) CV curves of (a, b) SC-0.5h, (c, d) SC-5h and (e, f) PyC anodes.


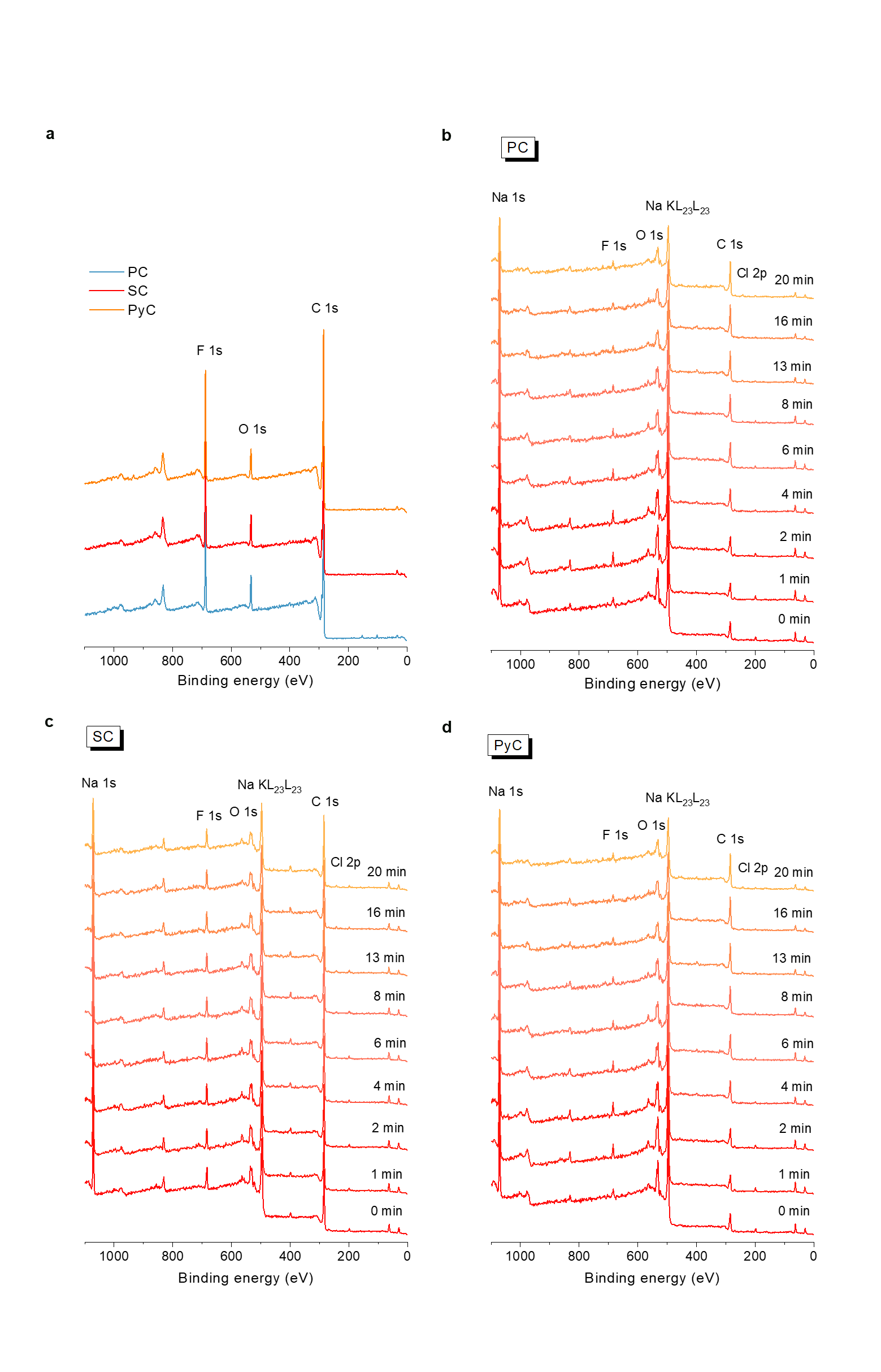


**Figure S12. In-depth XPS analysis of SEI species of PC, SC and PyC anodes.** (a) XPS survey spectra of the pristine PC, SC, and PyC anodes. Depth-dependent XPS survey spectra of (b) PC, (c) SC and (d) PyC anodes after 10 full cycles at a current density of 50 mA g^-1^.


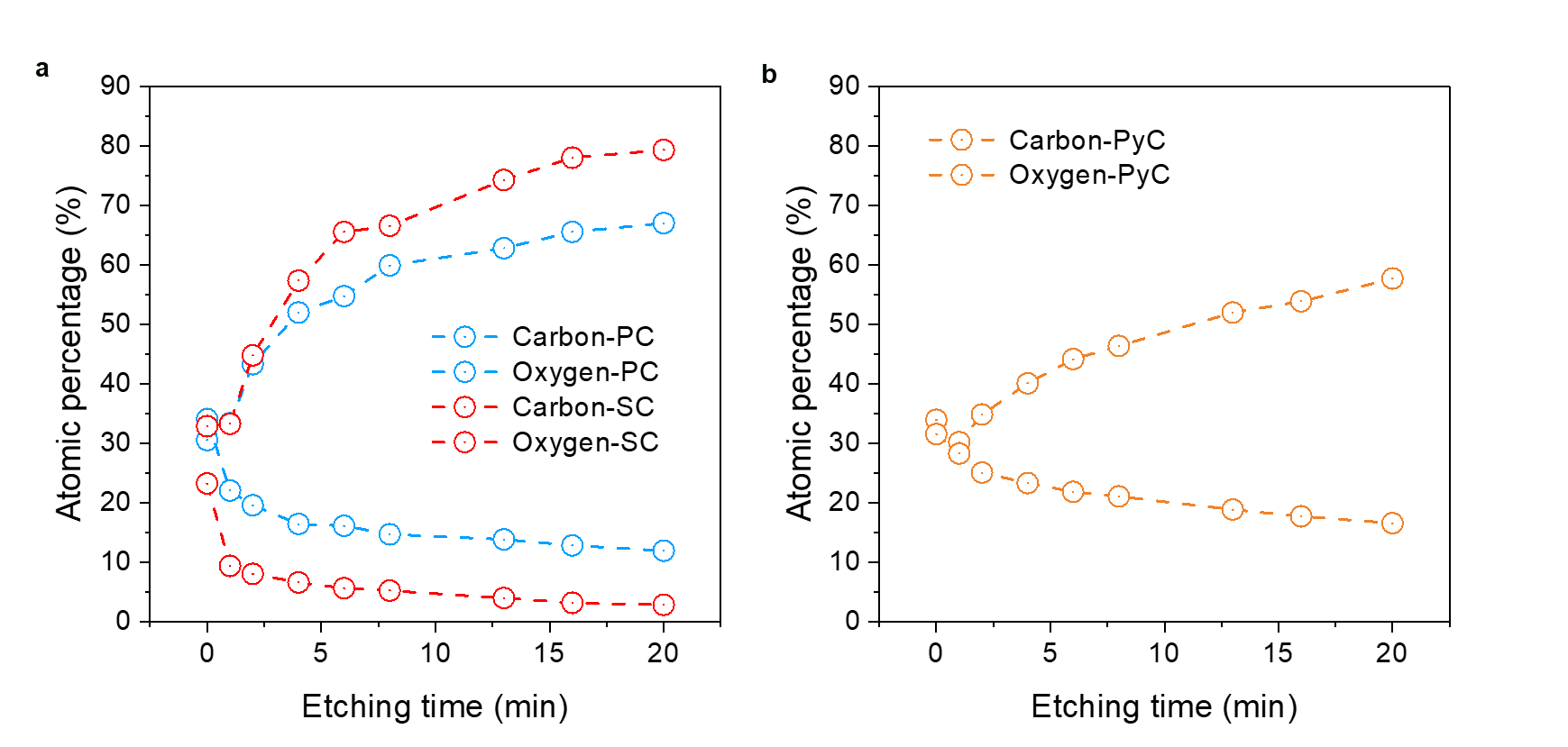


**Figure S13. Comparison of elemental percentages in PC, SC and PyC anodes.** Atomic percentage of carbon and oxygen in (a) PC, SC and (b) PyC anodes after 10 full cycles at a current density of 50 mA g^-1^.


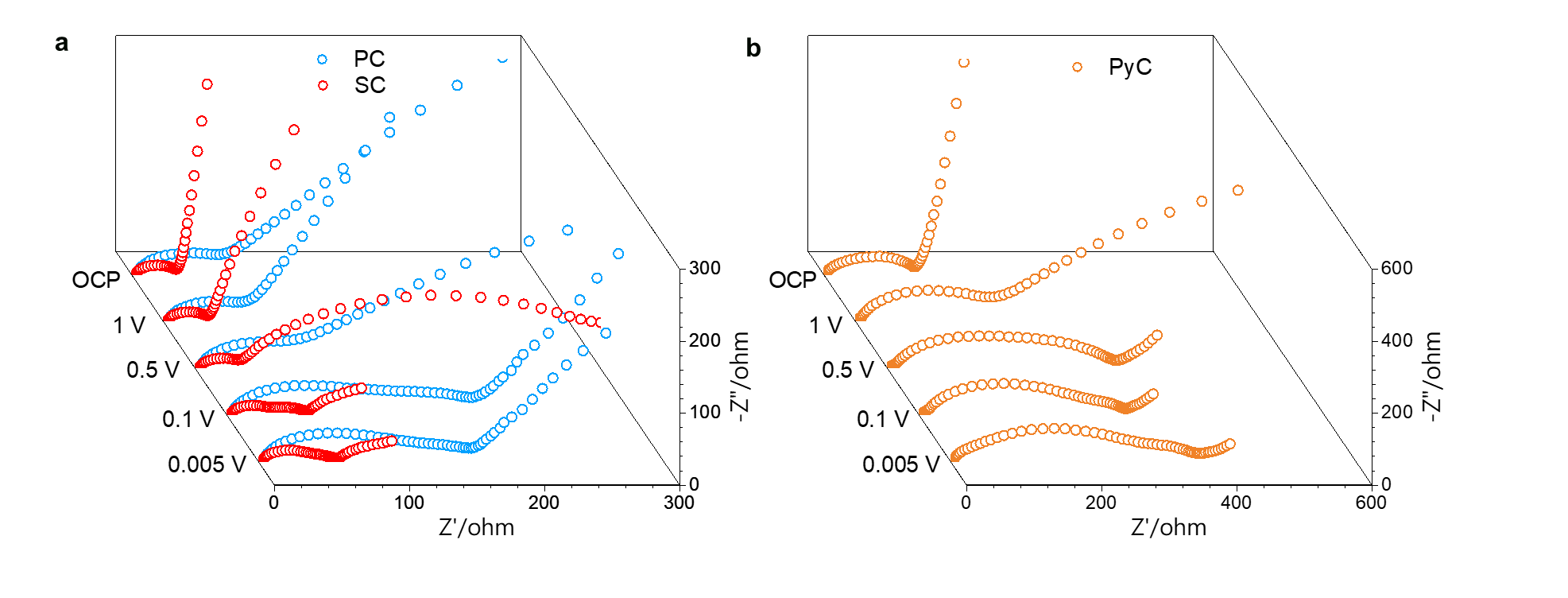


**Figure S14. *In-situ* EIS studies of PC, SC and PyC anodes.** EIS of (a) PC, SC and (b) PyC anodes at various states of charge (SOCs) during sodiation in the 1^st^ cycle.


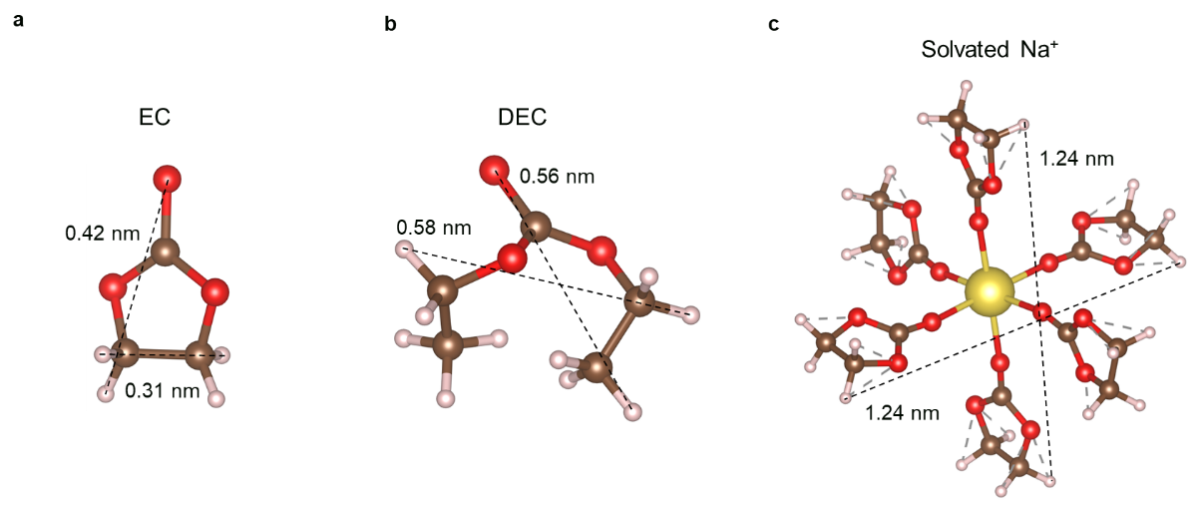


**Figure S15.** Schematics of the molecular structures of (a) EC, (b) DEC, and (c) solvated Na^+^ (with EC).


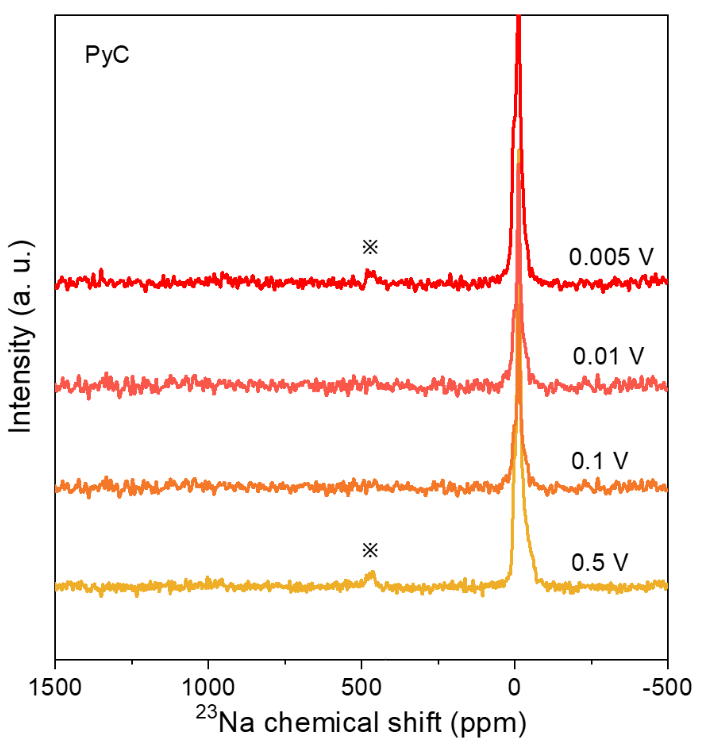


**Figure S16.** *Ex-situ* ^23^Na magic-angle-spinning (MAS) solid-state nuclear magnetic resonance (ssNMR) spectra of the PyC anode at various states of discharge in the 1^st^ cycle. The spinning sideband is labelled with an asterisk (※).


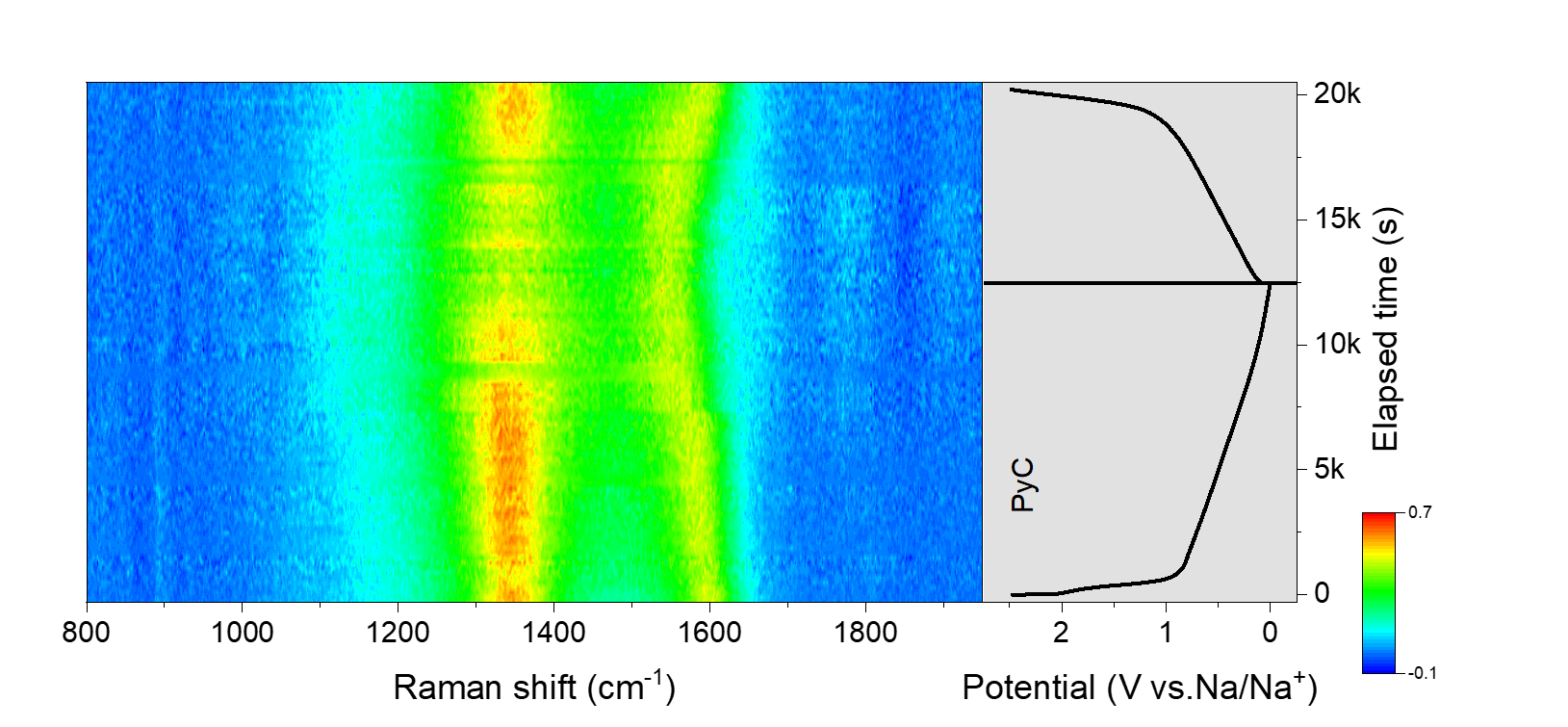


**Figure S17.** *Operando* Raman spectrum of a PyC anode in the 1^st^ cycle and the corresponding charge/discharge profiles at a rate of 50 mA g^-1^.


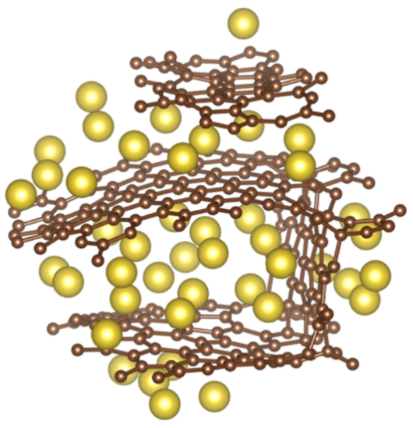


**Figure S18.** Structural snapshot of sodium atoms inside the model carbon (taking Na_44_C_200_ as an example). The model carbon is generated with 200 atoms per cell and the mass density is 1.153 g cm^-3^. Na atoms are large yellow spheres and carbon atoms are smaller blown spheres.


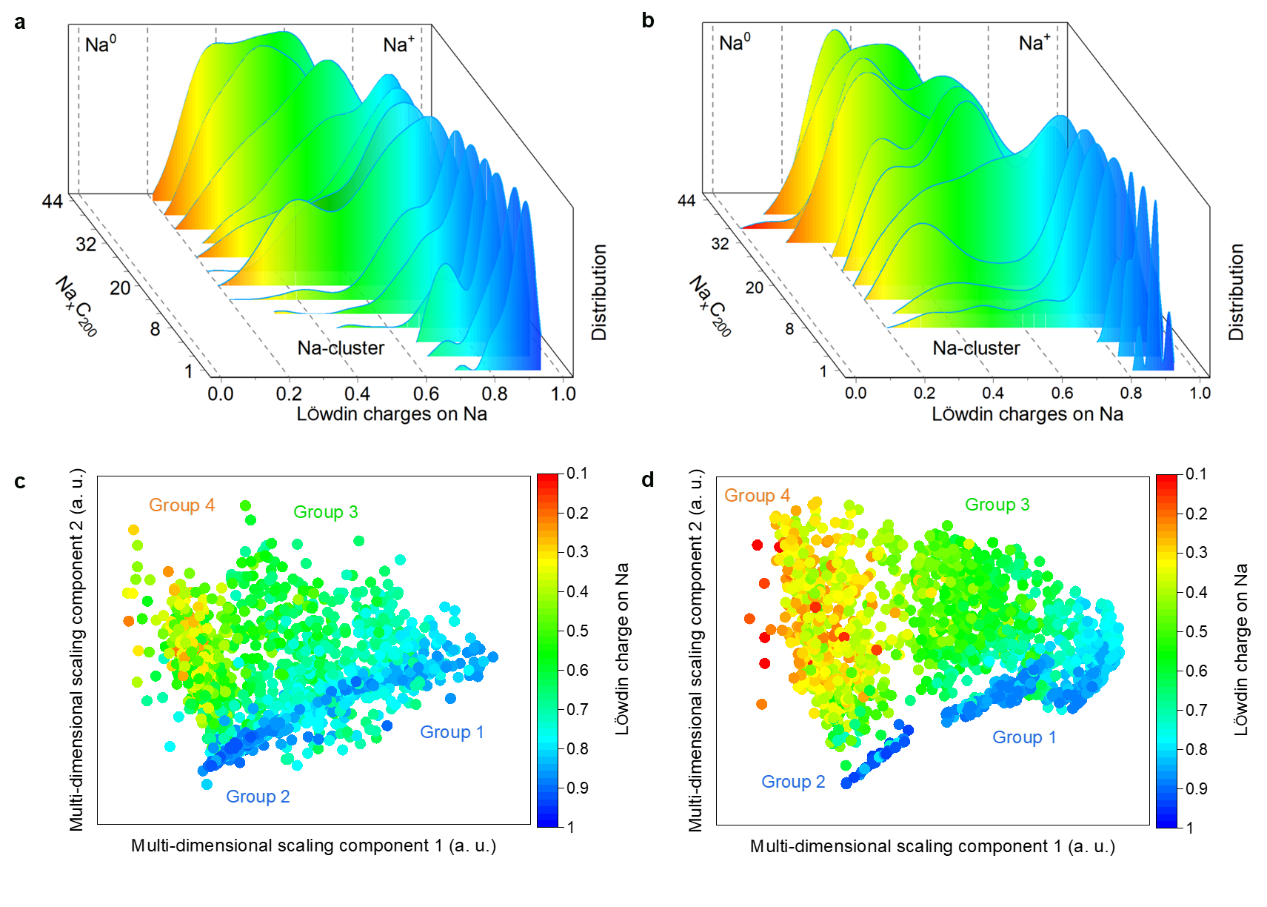


**Figure S19. Theoretical insight into dynamic sodium clustering process in SCs with different pore body diameters.** (a, b) Löwdin charges for Na atoms with increased filling from Na_1_C_200_ to Na_44_C_200_, drawn as kernel density estimated from the smoothed histograms, and (c, d) local environments of sodium storage analyzed using the SOAP kernel in the model carbon with (a, c) a small (~0.7 nm) and (b, d) a large pore body diameter (~0.9 nm). The model carbons with pore body diameters of approximately 0.7 nm and 0.9 nm were generated with 200 atoms per cell while keeping the shortest-path ring statistics fixed by regulating the mass densities to 0.699 and 1.491 g cm^-3^, respectively.


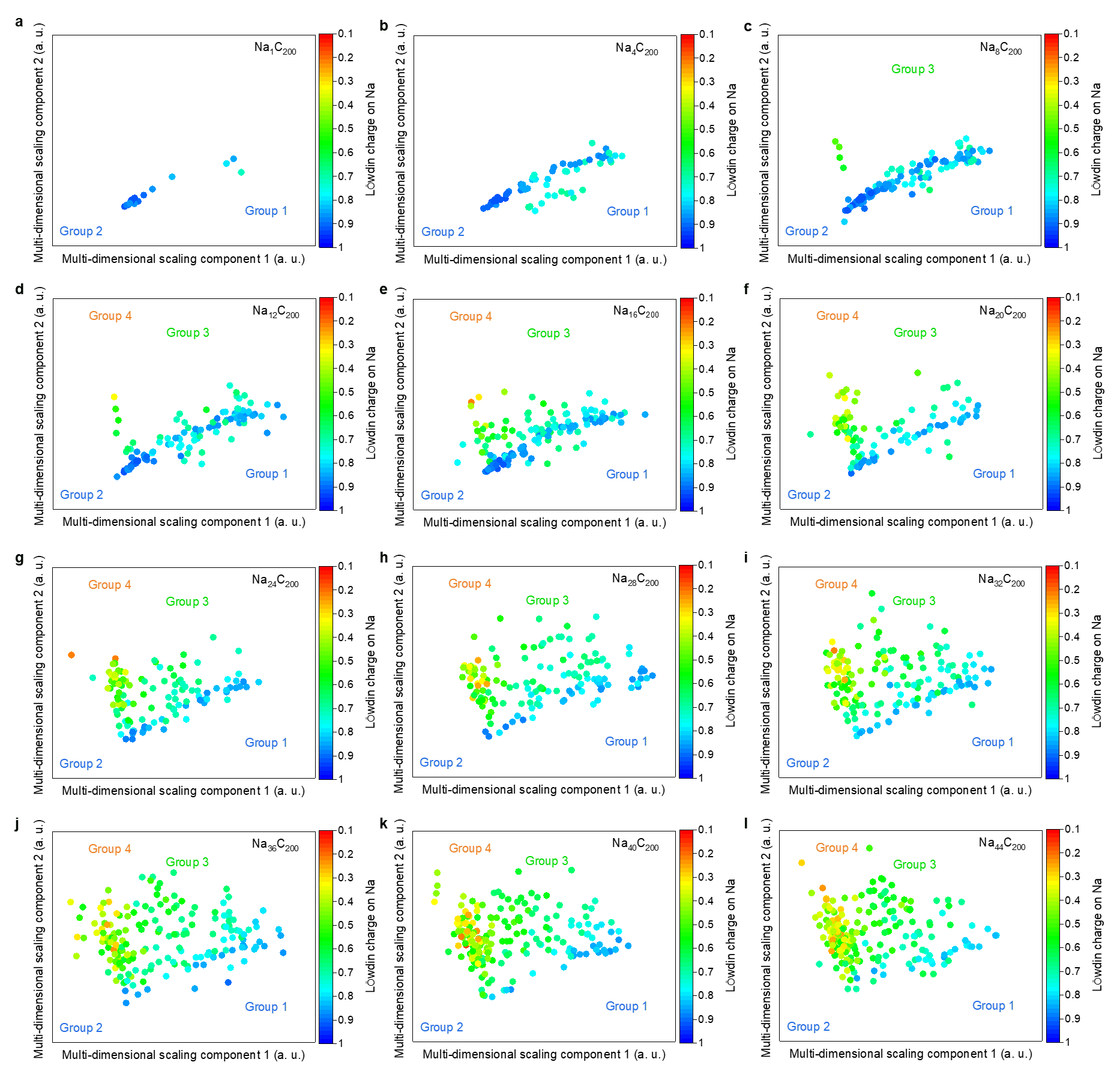


**Figure S20. Local environment analysis of sodium stored in a model carbon with a small pore body diameter (approximately 0.7 nm in the structural snapshot).** The corresponding model carbon was generated with a mass density of 0.699 g cm^-3^. SOAP maps were obtained by multidimensional scaling based on the structural distances for different sodium concentrations including (a) Na^2^C_200_, (b) Na^4^C^200^, (c) Na^8^C^200^, (d) Na^12^C^200^, (e) Na^16^C^200^, (f) Na^20^C^200^, (g) Na^24^C^200^, (h) Na^28^C^200^, (i) Na^32^C^200^, (j) Na^36^C^200^, (k) Na^40^C^200^ and (l) Na^44^C^200^. The most similar points are aggregated with similar colors.


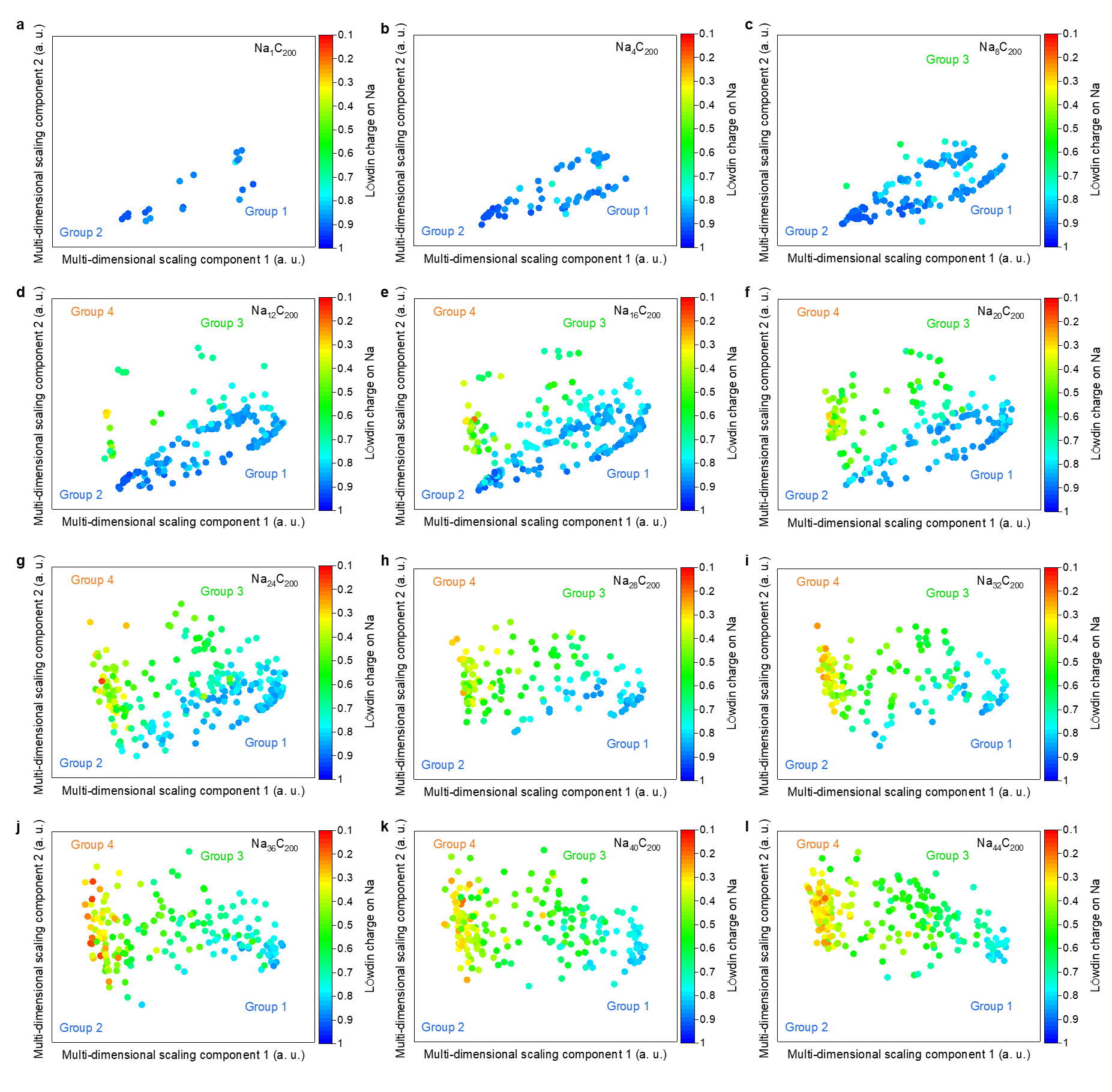


**Figure S21. Local environment analysis of sodium stored in a model carbon with a medium pore body diameter (approximately 0.8 nm in the structural snapshot).** The corresponding model carbon is generated with mass density of 1.153 g cm^-3^. SOAP maps were obtained by multidimensional scaling based on the structural distances at different sodium concentrations including (a) Na^2^C_200_, (b) Na^4^C^200^, (c) Na^8^C^200^, (d) Na^12^C^200^, (e) Na^16^C_200_, (f) Na^20^C^200^, (g) Na^24^C^200^, (h) Na^28^C^200^, (i) Na^32^C^200^, (j) Na^36^C^200^, (k) Na^40^C^200^ and (l) Na^44^C^200^. The most similar points are aggregated with similar colors.


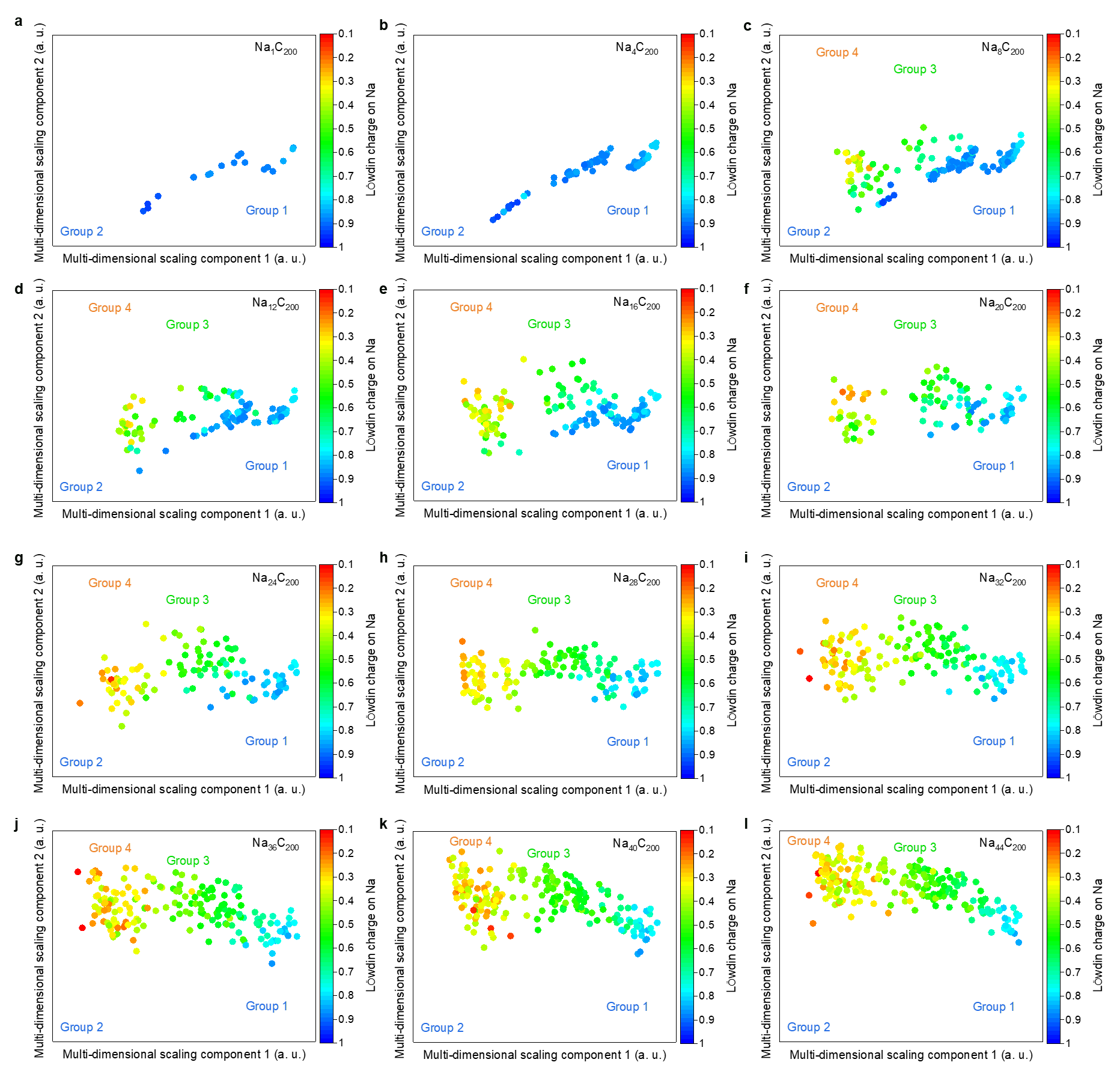


**Figure S22. Local environments analysis of sodium stored in a model carbon with a large pore body diameter (approximately 0.9 nm in the structural snapshot).** The corresponding model carbon was generated with a mass density of 1.491 g cm^-3^. SOAP maps were obtained by multidimensional scaling based on the structural distances at different sodium concentrations including (a) Na^2^C_200_, (b) Na^4^C^200^, (c) Na^8^C^200^, (d) Na^12^C^200^, (e) Na^16^C^200^, (f) Na^20^C^200^, (g) Na^24^C^200^, (h) Na^28^C^200^, (i) Na^32^C^200^, (j) Na^36^C^200^, (k) Na^40^C^200^ and (l) Na^44^C^200^. The most similar points are aggregated with similar colors.


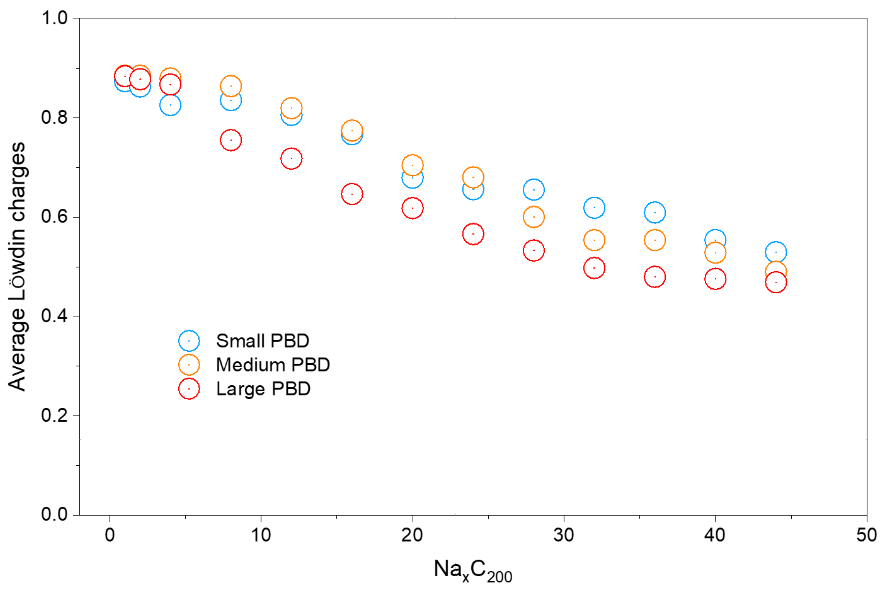


**Figure S23.** Average Löwdin charges of a single Na atom together with the degree of filling calculated by three models with different pore body diameters.


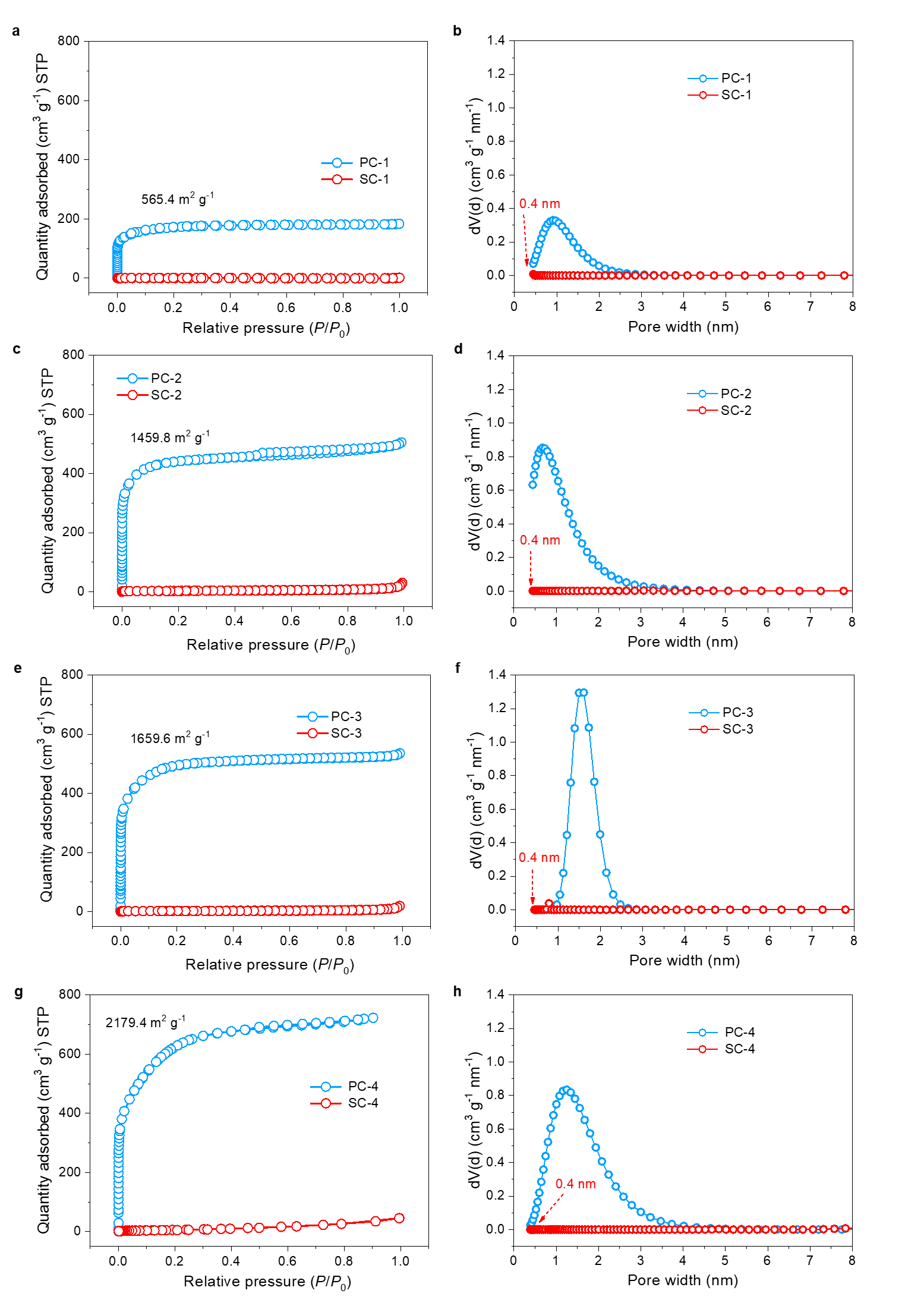


**Figure S24. Pore structure of** **PCs and SCs.** N^2^ adsorption-desorption isotherms at 77K of (a) SC-1, (c) SC-2, (e) SC-3 and (g) SC-4. The pore size distributions of (b) PC-1 and SC-1, (d) PC-2 and SC-2, (f) PC-3 and SC-3, and (h) PC-4 and SC-4.


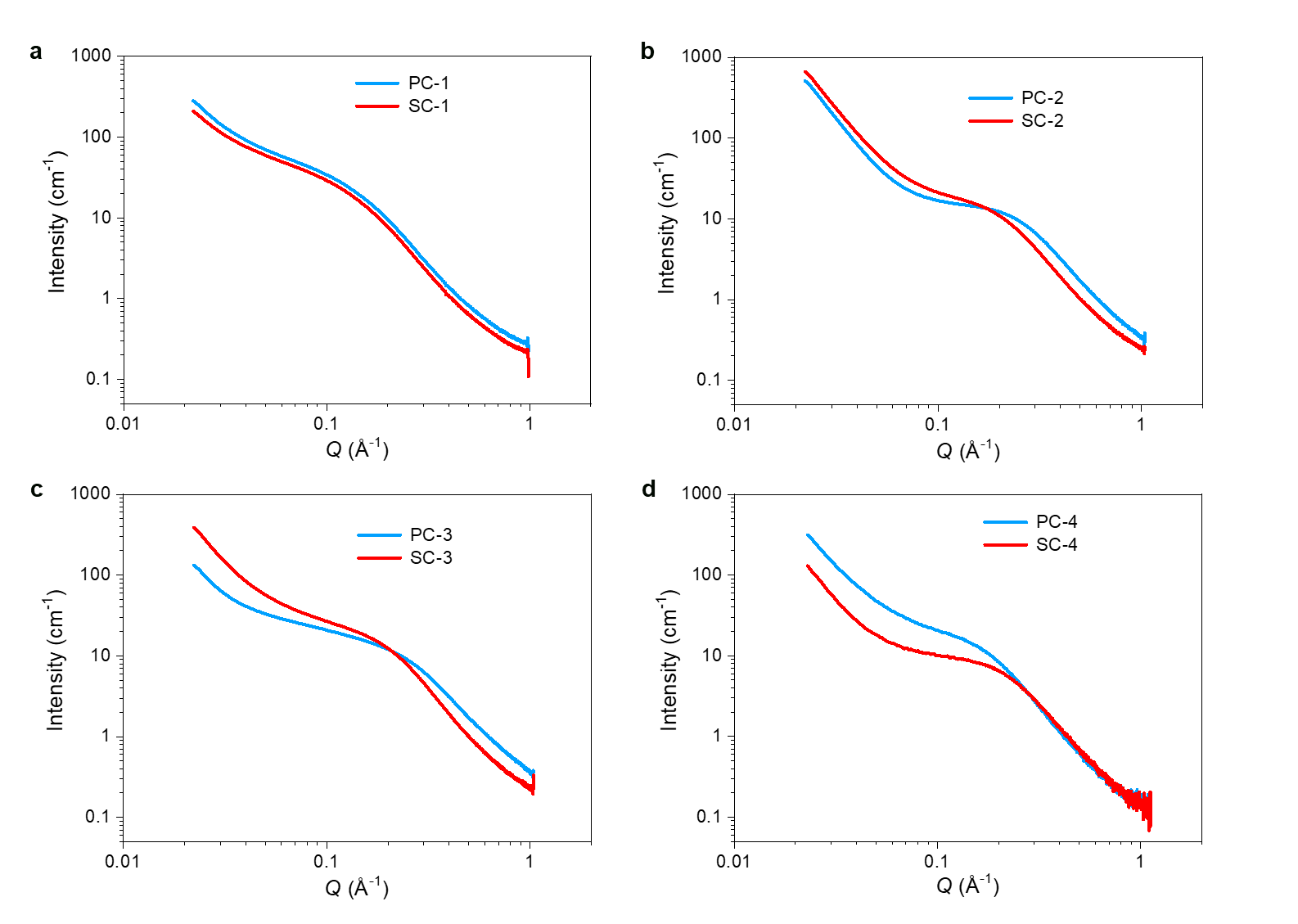


**Figure S25.** SAXS patterns of (a) PC-1 and SC-1, (b) PC-2 and SC-2, (c) PC-3 and SC-3, and (d) PC-4 and SC-4.


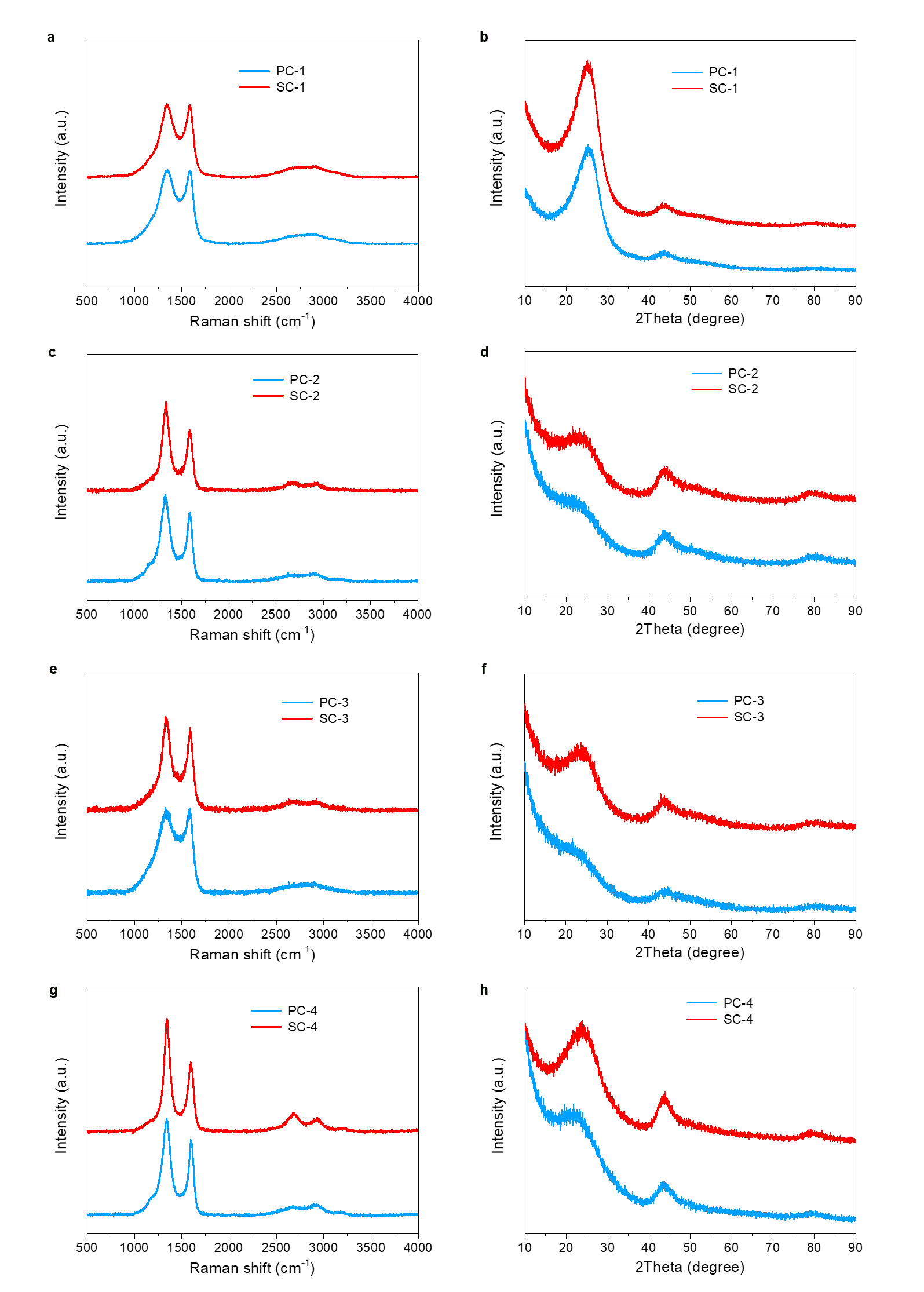


**Figure S26. Structural characterization of PCs and SCs.** (a, c, e, g) Raman spectra and (b, d, f, h) XRD patterns of (a, b) PC-1 and SC-1, (c, d) PC-2 and SC-2, (c, f) PC-3 and SC-3, and (g, h) PC-4 and SC-4.


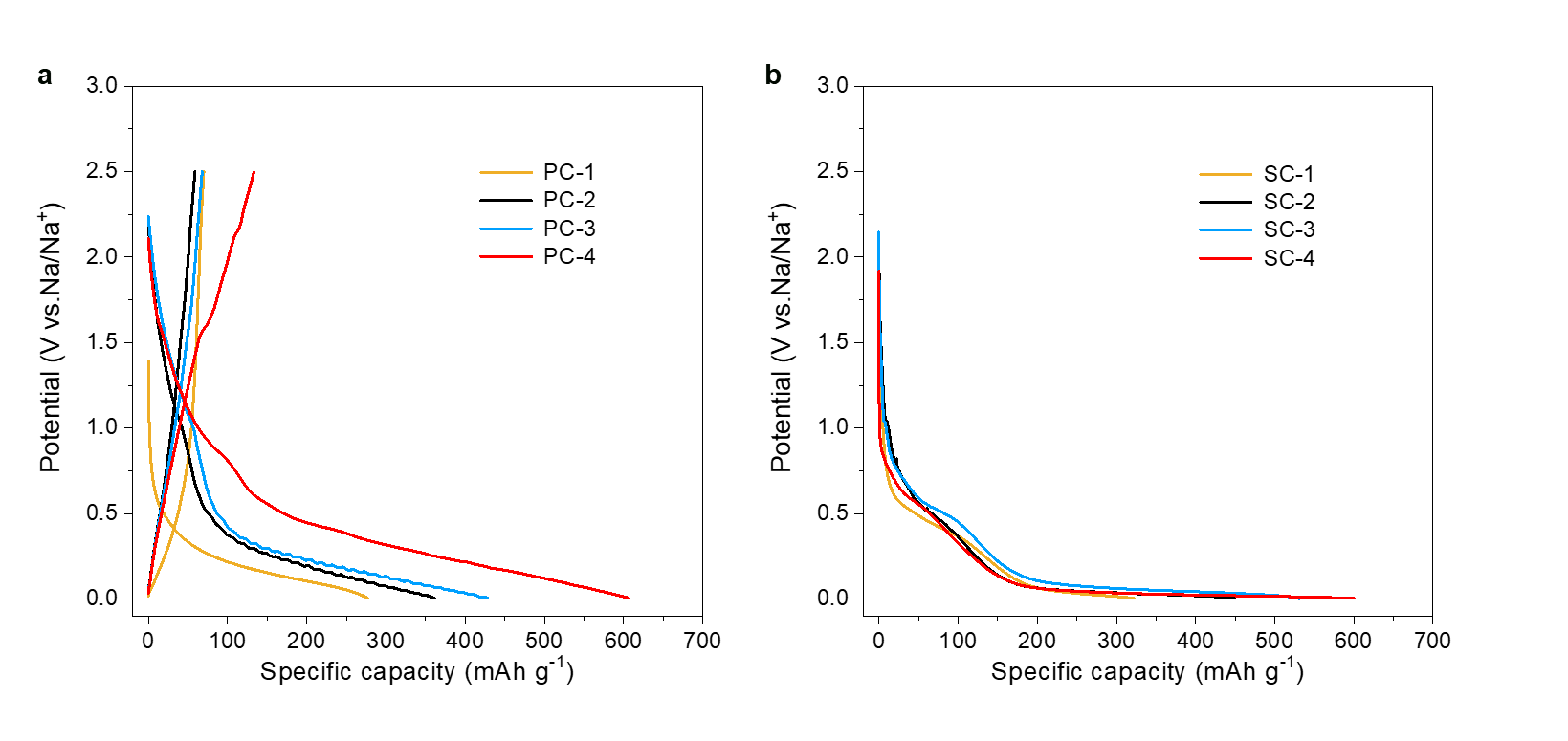


**Figure S27. Electrochemical performance of PC and SC anodes.** (a) Charge/discharge curves of different PC anodes, and (b) discharge curves of different SC anodes for the 1^st^ cycle at a current rate of 50 mA g^-1^.


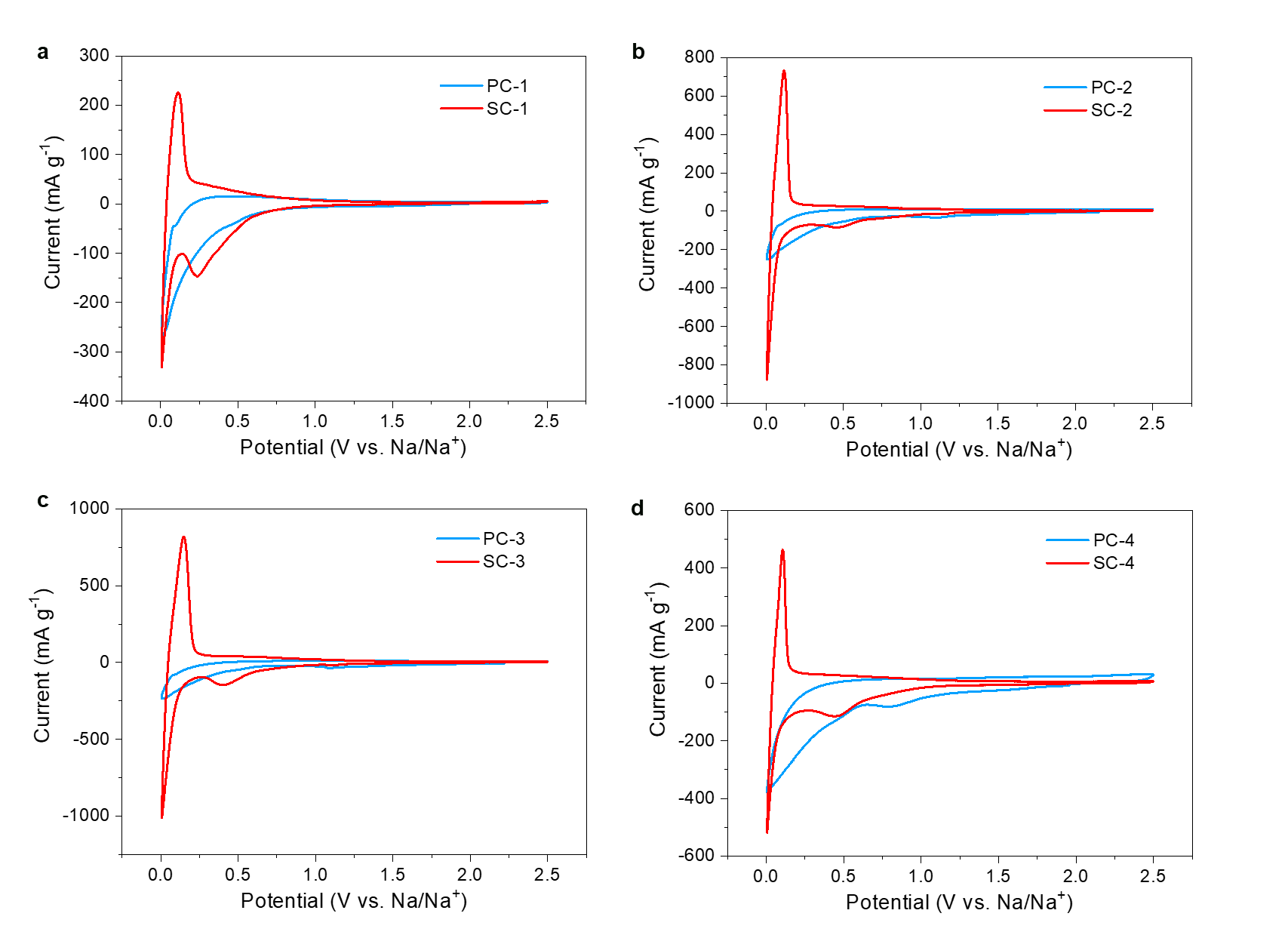


**Figure S28.** CV curves for the 1^st^ cycle at a scan rate of 0.1 mV s^-1^ of (a) PC-1 and SC-1, (b) PC-2 and SC-2, (c) PC-3 and SC-3, and (d) PC-4 and SC-4 anodes.


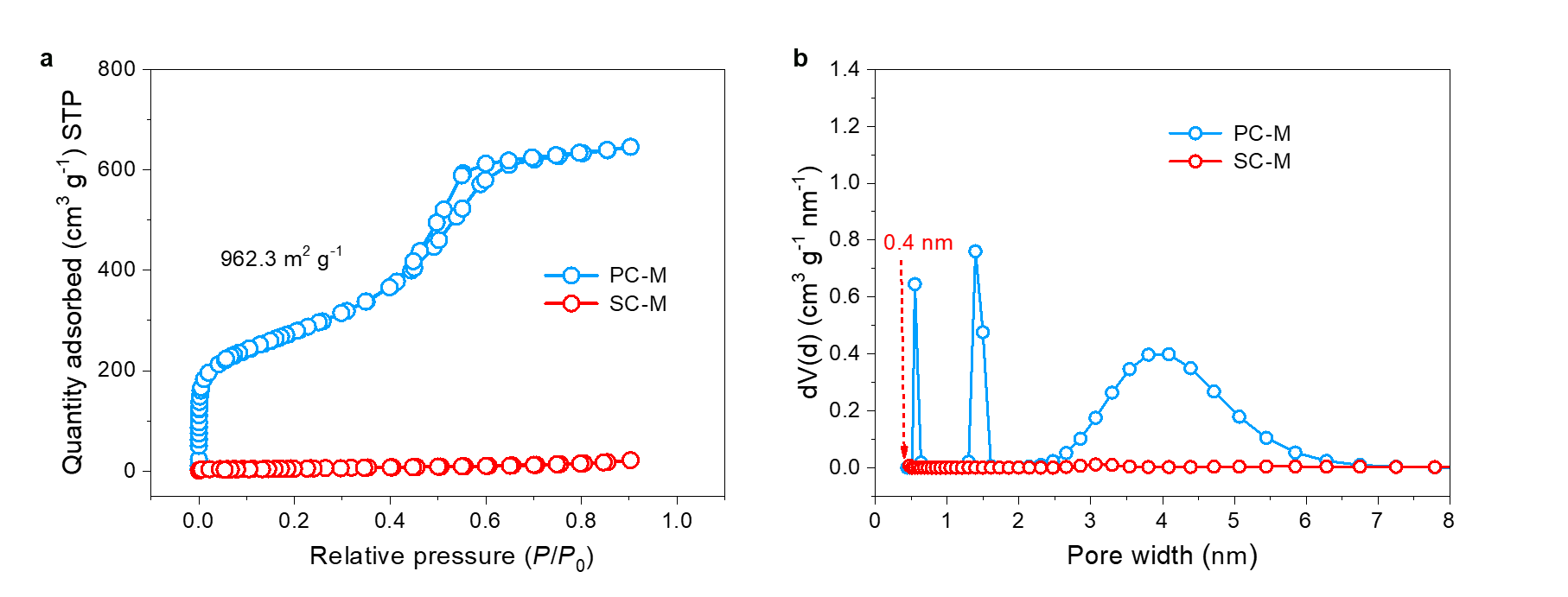


**Figure S29. Pore structure characterization of** **PC-M and SC-M.** (a) N_2_ adsorption-desorption isotherms at 77K, and (b) the corresponding pore diameter distributions of PC-M and SC-M.


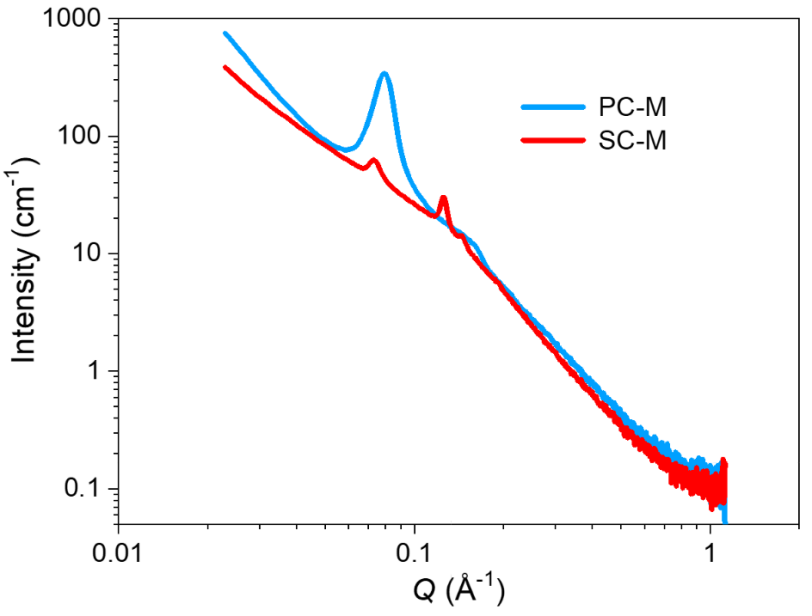


**Figure S30.** SAXS patterns of PC-M and SC-M.


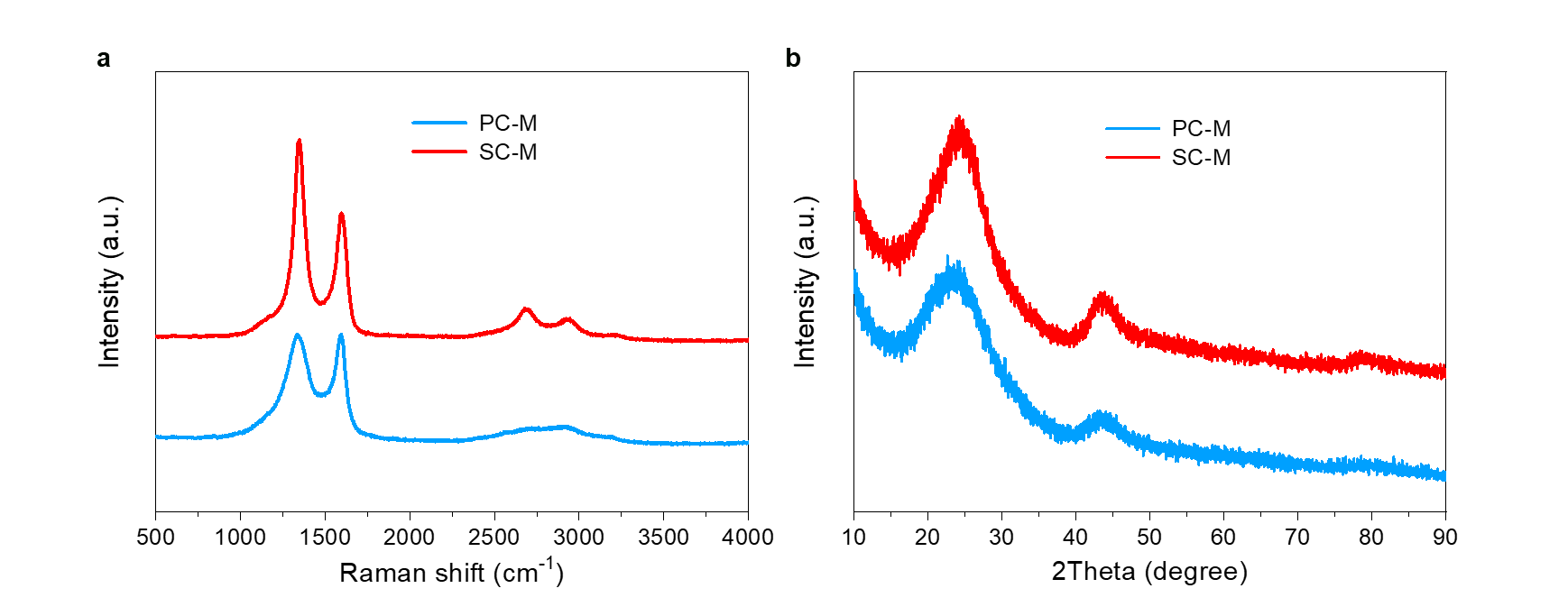


**Figure S31. Structural characterization of PC-M and SC-M.** (a) Raman spectra and (b) XRD patterns of PC-M and SC-M.


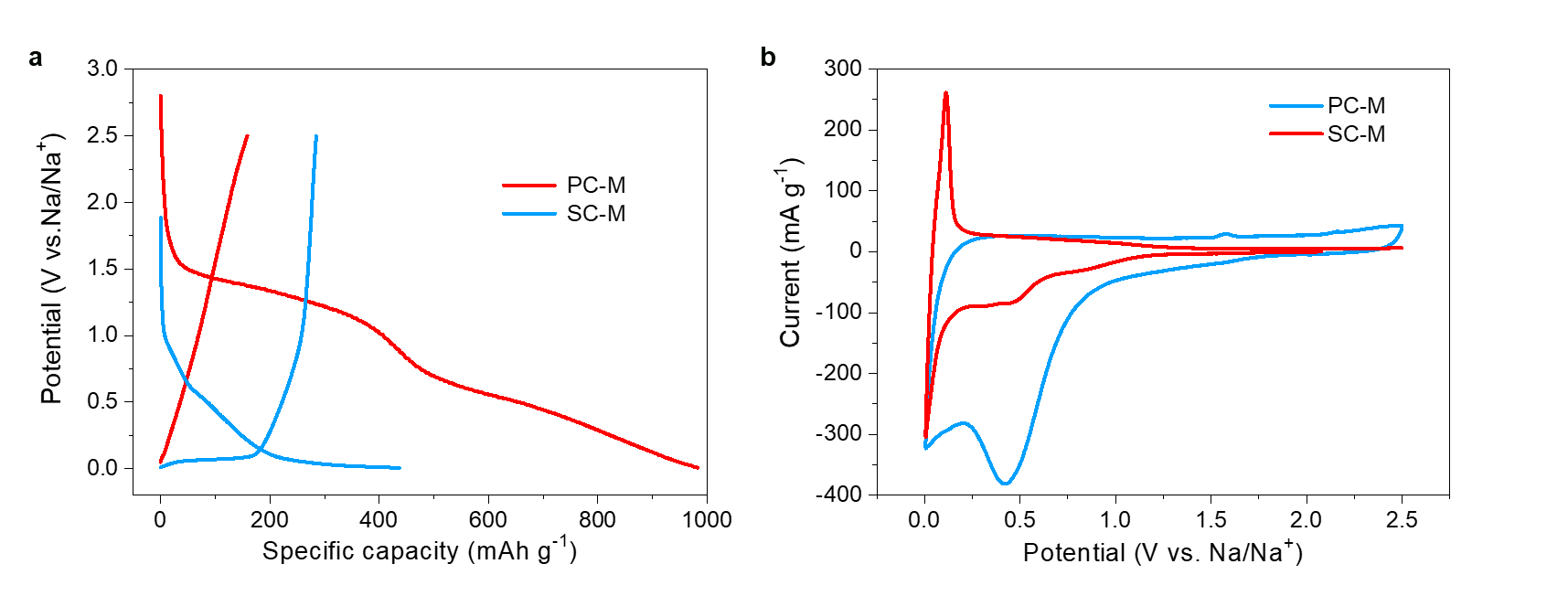


**Figure S32. Electrochemical characterization of PC-M and SC-M anodes.** (a) 1^st^- cycle charge/discharge curves at a current density of 50 mA g^-1^ and (b) CV curves at a scan rate of 0.1 mV s^-1^ of PC-M and SC-M anodes.


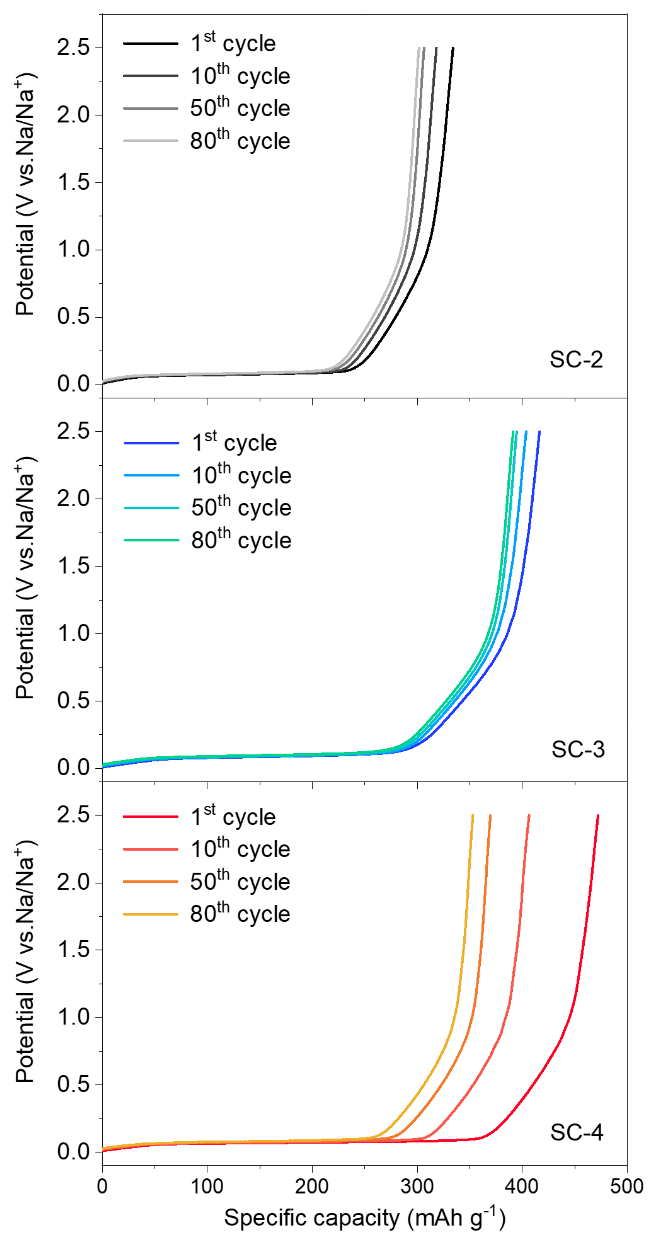


**Figure S33.** Charge curves in different cycles at a current density of 50 mA g^-1^ for SC-2, SC-3, and SC-4 anodes.


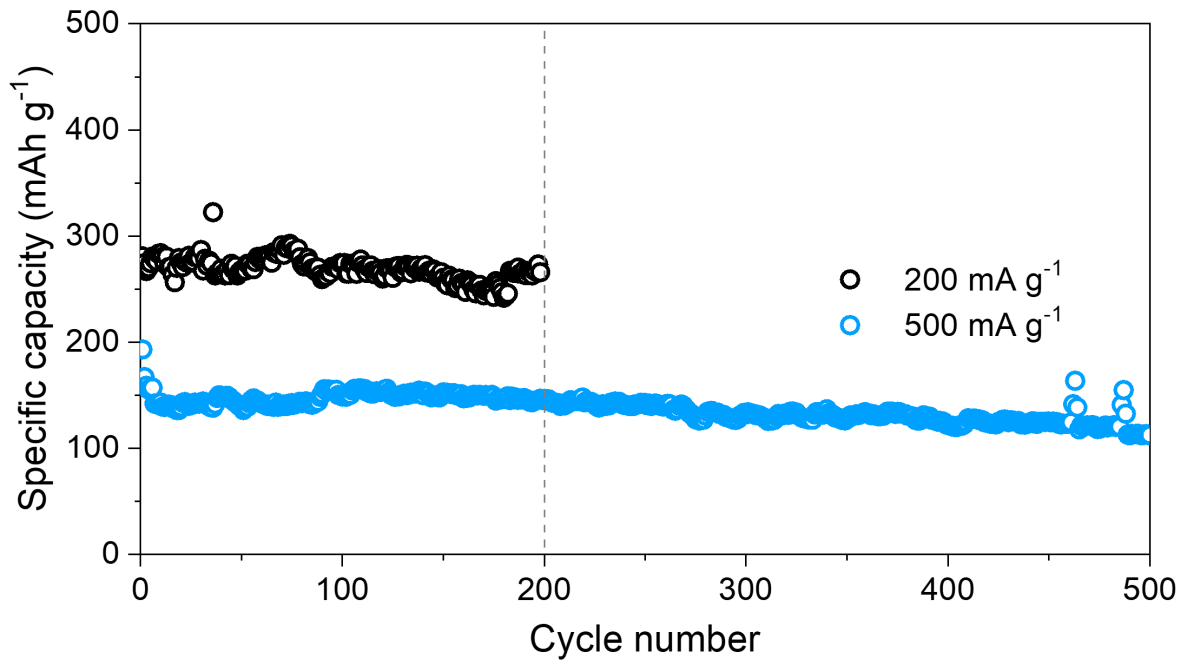


**Figure S34.** Cycling performance of a SC-3 anode at current densities of 200 and 500 mA g^-1^.

**Table S1. Physical parameters of the obtained carbons.**

| Samples | SSA^N2^  (m^2^ g^-1^) | V^N2^  (cm^3^ g^-1^) | d_002_  (nm) | L_c_  (nm) | I_G_/I_D_^a)^ | L_a_^a)^  (nm) | I_G_/I_D_^b)^ | L_a_^b)^  (nm) | PyC content (wt. %) |
| --- | --- | --- | --- | --- | --- | --- | --- | --- | --- |
| PyC | - | - | 0.361 | 1.71 | 0.80 | 9.59 | 0.80 | 24.06 | - |
| PC | 1358.0 | 0.59 | 0.389 | 1.18 | 0.34 | 6.56 | 1.26 | 15.19 | 0 |
| SC-0.5h | 419.0 | 0.26 | 0.388 | 1.20 | 0.30 | 5.66 | - | - | 9 |
| SC | - | - | 0.387 | 1.21 | 0.26 | 5.07 | 1.22 | 15.74 | 20 |
| SC-5h | - | - | 0.385 | 1.24 | 0.28 | 5.28 | - | - | 23 |
| PC-1 | 565.4 | 0.35 | 0.359 | 1.44 | 0.71 | 13.70 | - | - | 0 |
| SC-1 | - | - | 0.359 | 1.47 | 0.63 | 12.20 | - | - | 6 |
| PC-2 | 1459.8 | 0.94 | 0.384 | 1.13 | 0.36 | 8.70 | - | - | 0 |
| SC-2 | 11.2 | 0.06 | 0.373 | 1.44 | 0.29 | 7.23 | - | - | 34 |
| PC-3 | 1659.6 | 0.92 | 0.378 | 1.24 | 0.59 | 11.24 | - | - | 0 |
| SC-3 | 7.9 | 0.06 | 0.372 | 1.27 | 0.54 | 10.39 | - | - | 38 |
| PC-4 | 2179.4 | 1.29 | 0.378 | 1.11 | 0.48 | 9.29 | - | - | 0 |
| SC-4 | - | - | 0.372 | 1.18 | 0.45 | 8.70 | - | - | 47 |
| PC-M | 962.3 | 1.13 | 0.368 | 1.03 | 0.67 | 12.99 | - | - | 0 |
| SC-M | 19.8 | 0.03 | 0.365 | 1.16 | 0.43 | 8.18 | - | - | 61 |

^a)^ I_G_/I_D_ and L_a_ is calculated by Raman with wavelength of 532 nm.

^b)^ I_G_/I_D_ and L_a_ is calculated by Raman with wavelength of 325 nm.

**Table S2. Morphological parameters of the nanopores deduced from SAXS patterns based on the Teubner-Strey model of the obtained carbons.**

| Samples | Structure density  (g cm^-3^) | ΔSLD  (*10^-6^ Å^-2^) | d (nm) | ξ (nm) | D (nm) | SSA^SAXS^  (m^2^ g^-1^) |
| --- | --- | --- | --- | --- | --- | --- |
| PC | 2.39 | 20.4 | 6.20 | 0.37 | 2.00 | 1313 |
| SC | 2.32 | 20.1 | 6.20 | 0.37 | 2.00 | 1298 |
| PC-1 | 2.27 | 20.4 | 4.8 | 0.68 | 1.97 | 338 |
| SC-1 | 2.23 | 20.0 | 4.8 | 0.68 | 1.96 | 330 |
| PC-2 | 2.32 | 19.7 | 6.3 | 0.40 | 2.00 | 1882 |
| SC-2 | 2.27 | 19.3 | 5.6 | 0.53 | 1.99 | 1447 |
| PC-3 | 2.22 | 18.9 | 6.4 | 0.37 | 1.94 | 2360 |
| SC-3 | 2.27 | 19.3 | 5.4 | 0.50 | 1.90 | 1786 |
| PC-4 | 2.32 | 19.8 | 8.0 | 0.50 | 2.52 | 2538 |
| SC-4 | 2.27 | 19.3 | 6.8 | 0.60 | 2.41 | 2059 |
| PC-M | 2.26 | 19.2 | 11.0 | 0.97 | 3.91 | 1283 |
| SC-M | 2.25 | 19.1 | 10.0 | 0.94 | 3.56 | 1080 |

**Table S3. Electrochemical properties of the obtained carbon anodes.**

| Samples | Discharge capacity (mAh g^-1^) | Charge capacity  (mAh g^-1^) | Irreversible capacity  (mAh g^-1^) | ICE  (%) | Plateau capacity  (mAh g^-1^) |
| --- | --- | --- | --- | --- | --- |
| PyC | 307 | 167 | 140 | 54 | 0 |
| PC | 259 | 39 | 220 | 15 | 0 |
| SC-0.5h | 322 | 156 | 165 | 49 | 89 |
| SC | 425 | 328 | 97 | 77 | 241 |
| SC-5h | 395 | 272 | 123 | 69 | 156 |
| PC-1 | 277 | 70 | 207 | 28 | 0 |
| SC-1 | 322 | 183 | 139 | 57 | 110 |
| PC-2 | 361 | 59 | 302 | 16 | 0 |
| SC-2 | 450 | 336 | 113 | 75 | 266 |
| PC-3 | 428 | 68 | 360 | 16 | 0 |
| SC-3 | 531 | 430 | 101 | 81 | 285 |
| PC-4 | 766 | 147 | 620 | 19 | 0 |
| SC-4 | 600 | 482 | 118 | 80 | 400 |
| PC-M | 983 | 158 | 825 | 16 | 0 |
| SC-M | 437 | 285 | 152 | 65 | 198 |

**REFERENCES**

[1] Saurel, D, Segalini, J, Jauregui, M*, et al.* A SAXS outlook on disordered carbonaceous materials for electrochemical energy storage. *Energy Storage Mater*. 2019; **21**: 162-73.

[2] Huang, JX, Csanyi, G, Zhao, JB*, et al.* First-principles study of alkali-metal intercalation in disordered carbon anode materials. *J Mater Chem A*. 2019; **7**: 19070-80.

[3] Urban, A, Seo, D-H, Ceder, G. Computational understanding of Li-ion batteries. *NPJ Comput Mater*. 2016; **2**.

[4] Schubert, KV, Strey, R, Kline, SR*, et al.* Small angle neutron scattering near Lifshitz lines: Transition from weakly structured mixtures to microemulsions. *J Chem Phys*. 1994; **101**: 5343-55.

[5] Teubner, M, Strey, R. Origin of the scattering peak in microemulsions. *J Chem Phys*. 1987; **87**: 3195-200.

[6] Porod, G. General theory. *Small angle X-ray scattering*. 1982.

[7] Stevens, DA, Dahn, JR. The mechanisms of lithium and sodium insertion in carbon materials. *J Electrochem Soc*. 2001; **148**: A803.

[8] Jeromenok, J, Weber, J. Restricted access: on the nature of adsorption/desorption hysteresis in amorphous, microporous polymeric materials. *Langmuir*. 2013; **29**: 12982-9.

[9] Nguyen, C, Do, D. Preparation of carbon molecular sieves from macadamia nut shells. *Carbon*. 1995; **33**: 1717-25.

[10] Ferrari, AC, Robertson, J. Interpretation of Raman spectra of disordered and amorphous carbon. *Phys Rev B*. 2000; **61**: 14095-107.

[11] Ferrari, AC, Robertson, J. Resonant Raman spectroscopy of disordered, amorphous, and diamondlike carbon. *Phys Rev B*. 2001; **64**: 13.

[12] Hardwick, LJ, Ruch, PW, Hahn, M*, et al.* In situ Raman spectroscopy of insertion electrodes for lithium-ion batteries and supercapacitors: First cycle effects. *J Phys Chem Solids*. 2008; **69**: 1232-7.

[13] Yamauchi, S, Kurimoto, Y. Raman spectroscopic study on pyrolyzed wood and bark of Japanese cedar: temperature dependence of Raman parameters. *J Wood Sci*. 2003; **49**: 235-40.

[14] Zickler, GA, Smarsly, B, Gierlinger, N*, et al.* A reconsideration of the relationship between the crystallite size La of carbons determined by X-ray diffraction and Raman spectroscopy. *Carbon*. 2006; **44**: 3239-46.

[15] Kawabuchi, Y, Kishino, M, Kawano, S*, et al.* Carbon deposition from benzene and cyclohexane onto active carbon fiber to control its pore size. *Langmuir*. 1996; **12**: 4281-5.

[16] Dopita, M, Rudolph, M, Salomon, A*, et al.* Simulations of X-ray scattering on two-dimensional, graphitic and turbostratic carbon structures. *Adv Eng Mater*. 2013; **15**: 1280-91.
